# Thirty-Sixth Annual Meeting of the Society for Light, Rhythms, and Circadian Health (SLRCH), 14–16 June, Boston, MA, USA

**DOI:** 10.3390/clockssleep8010002

**Published:** 2025-12-31

**Authors:** Corrado Garbazza

**Affiliations:** Centre for Chronobiology, University of Basel, 4002 Basel, Switzerland; corrado.garbazza@upk.ch

## 1. Introduction

It is my pleasure to present this collection of abstracts from the 36th Annual Meeting of the Society for Light, Rhythms, and Circadian Health (SLRCH), held in Boston, Massachusetts, at Simmons University and Brigham and Women’s Hospital. This year’s meeting marked another milestone in our society’s mission to advance the understanding of light, rhythms, and circadian health, with an inspiring blend of cutting-edge science, vibrant discussion, and collaborative spirit.

Our program reflected the breadth and vitality of the field, spanning basic, translational, and clinical research. Highlights included oral and poster presentations on topics ranging from circadian medicine, mood and neurodegenerative disorders, sleep interventions, chrononutrition, and light-based technologies, to innovative modeling approaches. The “Year in Review” session once again captured the rapid progress across basic, translational, clinical, and technological domains.

We were especially honored to hear the keynote lecture by Charles A. Czeisler, PhD, MD, whose presentation on the impact of natural and artificial light on human health set a compelling tone for the meeting. In addition, invited symposia and oral sessions showcased exciting work in circadian medicine, neurobiology, aging, adolescent sleep health, genetics, and spaceflight lighting research.

This year’s program also featured a successful Trainee Day, an engaging Industry Symposium, and stimulating poster sessions that created opportunities for early-career and established researchers alike to exchange ideas and build collaborations. The energy and enthusiasm in Boston were tangible, underscoring the importance of our collective efforts to advance circadian health in science, medicine, and society.

I wish to extend my heartfelt thanks to our board members, session chairs, presenters, sponsors, and our local hosts in Boston for their generous hospitality and organizational excellence. Your dedication and support made this meeting a resounding success.

Please enjoy reading through these abstracts which capture the scientific vision, rigor, and innovation of our community. I am deeply grateful to all of you, scientists, clinicians, trainees, sponsors, and organizers, for contributing to the success of the 36th Annual Meeting and for advancing the mission of the SLRCH.

With warm regards,
**Corrado Garbazza, MD, PhD**
President, SLRCH

## 2. Real-World Light Exposure and Chronotype: Insights from Wearable Near-Corneal Sensors

**Authors**

Dr. Oliver Stefani-Switzerland-Lucern University of Applied Sciences and Arts

Dr. Denis Gubin-Russian Federation-Tyumen State Medical University

Mr. Reto Marek-Switzerland-Lucern University of Applied Sciences and Arts

Prof. Björn Schrader-Switzerland-Lucern University of Applied Sciences and Arts

**Abstract**

**Background:** Acceptance of wearable light loggers is critical for consistent use and reliable data collection. Uncomfortable or socially inconvenient devices reduce compliance and compromise data quality. Wrist-worn sensors, while less obtrusive, inaccurately capture corneal light exposure, which is critical for assessing non-image-forming (NIF) effects. This limitation is most significant in the evening, when small changes in ambient light can greatly affect NIF effects. **Methods:** 29 participants (17 women, aged 18–35 years) wore a light dosimeter (lido) at glasses for five days (during 8 October–4 December 2023). They then completed an online Qualtrics survey, including the Morning-Evening Questionnaire (MEQ). The lido recorded near corneal light exposure every 10 s using CIE S 026:2018 metrics. Data pre-processing identified sleep times using automated tilt analysis, light levels, user logs and motion-based sleep estimation. Daytime was defined as from waking to three hours before bedtime; evening was defined as the last three hours before sleep. **Results:** Participants wore lido for 89% of their waking hours. Morning types never wore it after midnight, while evening types never wore it before 8:30 am. While outdoor illuminance in Lucerne exceeded 250 lx mEDI from early morning, peaking at over 50,000 lx mEDI at midday, the median melanopic equivalent daylight illuminance (mEDI) at eye level was only 51 lx mEDI (mean: 299, max: 93,432), showing significant variability. Participants spent only 14% of their wearing time above 250 lx mEDI, indicating inadequate daytime light exposure. Most of the day was spent below 100 lx mEDI, indicating insufficient exposure to the light level recommendations. Evening exposure remained below 10 lx mEDI for 57% of wear time. Wake-up times varied significantly between weekdays and weekends (average delay: 1 h 36 min). We found a correlation between MEQ scores and the timing of light exposure over a five-day measurement period. Phase estimates derived from fitting a 24-h cosine function to the entire sampling period differed from those obtained when cosine functions were fitted separately for each weekday. Only phase estimates from Saturday data correlated with MEQ scores, whereas weekday data showed no such correlation, suggesting that light exposure during office work differs from that on weekends and may impede MEQ prediction. **Conclusions:** Near-corneal light exposure, as recorded by wearable light dosimeters, revealed substantial variability in daily and evening light exposure patterns. While the mean mEDI suggested adequate light exposure, the median indicated that most participants experienced low levels during the day. Participants’ limited exposure to recommended daytime light levels suggests a potential deficit in light-mediated circadian entrainment. Compliance rates were high, with differences in wear time observed between chronotypes. Our results demonstrate a correlation between MEQ scores and light exposure timing. Wearable light dosimeters provide valuable real-world data to inform public health strategies to optimize daily light exposure for well-being. Future research should refine compliance strategies and explore alternative measurement approaches to enhance the precision of non-image-forming light exposure assessments.

**Keywords:** wearable light logger; real-world corneal light exposure; chronotype

**Funding**: This work was funded by the Velux Foundation Switzerland.

## 3. Light Regularity and Sleep Patterns Among Adolescents with ADHD

**Author**

Dr. Jessica Lunsford-Avery-United States-Duke University School of Medicine

**Abstract**

**Background**: Light exposure may play a key role in the pathology of attention-deficit/hyperactivity disorder (ADHD) through its impact on circadian rhythm regulation. Adolescents with ADHD display irregular and delayed sleep patterns, which may also result from circadian dysfunction. Regularity of light exposure is related to sleep regularity and timing in adolescents generally; however, whether light exposure irregularity contributes to irregular/delayed sleep in adolescents with ADHD is unknown. Our primary goals were to investigate: (1) differences in light exposure regularity among adolescents with ADHD versus healthy controls without psychiatric disorders (HC) and (2) associations between light regularity and sleep regularity and timing, chronotype preference, and daytime sleepiness. **Methods:** Forty-six adolescents aged 13–17 (54% female, mean age = 15.25) completed a diagnostic interview, >5 days/nights of actigraphy (Actiwatch Spectrum Pro), and self-reports of chronotype and daytime sleepiness (Morningness-Eveningness Scale for Children, Cleveland Adolescent Sleepiness Questionnaire). Twenty were diagnosed with ADHD; 26 were healthy controls (HC). Linear regressions examined group differences in the light regularity index (LRI), derived from actigraph-measured light exposure. Partial Pearson correlations examined associations of the LRI with sleep measures (Sleep Regularity Index, Sleep Midpoint) in the full sample and the ADHD group specifically. Analyses covaried sex assigned at birth and age. **Results:** Adolescents with ADHD displayed lower LRI than HC, suggesting less regular light exposure in this group (β = 0.31, *p* = 0.04). In the full sample, lower LRI correlated with less regular (r = 0.44) and more delayed (r = −0.43) actigraph-measured sleep patterns and greater reported eveningness (r = 0.32) (*p*’s < 0.05); and daytime sleepiness at trend-level (r = −0.27, *p* = 0.07). In the ADHD group, the LRI was strongly associated with sleep regularity (r = 0.63) and timing (r = −0.50), *p*’s < 0.01; associations were non-significant in HC. **Conclusions:** Irregular light exposure may contribute to circadian dysfunction (irregular/delayed sleep) in adolescents with ADHD. Regularly-timed light exposure chronotherapy may improve sleep in youth with ADHD. Future studies using biological measures (e.g., dim-light melatonin onset) may clarify whether links between irregular light exposure and sleep disturbances in ADHD are mediated by the disruptive impact of light on circadian function.

**Keywords:** sleep regularity; sleep timing; light exposure; light regularity; adolescents; ADHD

**Funding:** K23-MH-108704; R34-MH-128440.

## 4. Light Exposure at Night and Obesity in Individuals with Schizophrenia: A Cross-Sectional Analysis of the LENS Study

**Authors**

Dr. Rina Taniguchi-Japan-Okehazama Hospital

Dr. Yuichi Esaki-Japan-Okehazama Hospital

Mr. Souji Tsuboi-Japan-Okehazama Hospital

Dr. Kiyoshi Fujita-Japan-Okehazama Hospital

**Abstract**

**Background:** Obesity and overweight are highly prevalent in individuals with schizophrenia and are associated with a risk of developing not only physical but also mental problems. Generally, light at night (LAN) exposure is associated with circadian disruption and metabolic troubles. However, a little study has investigated the association between LAN and obesity in individuals with schizophrenia. This cross-sectional study was to determine the relation between light exposure at night and obesity in schizophrenia. **Method:** Two-hundred and twenty-eight outpatients with schizophrenia were recruited to the Light Exposure and Neurobiology in Schizophrenia (LENS) study between March 2024 to November 2024. They were instructed to record bedroom light from bedtime to rising time using a portable photometer. They were also instructed to record all-day light exposure and activity by using an actigraph. LAN exposure in the bedroom was recorded at 1-min intervals. Furthermore, they were instructed to record their bedtime, and rising time. Body mass index (BMI) was determined using height and weight measured by the nurse at the hospital, and obesity was defined as a BMI ≥ 25 kg/m^2^. **Result:** The mean (standard deviation) weight, height, abdominal circumference, and BMI were 70.4 (14.5) kg, 1.63 (0.1) m, 94.3 (14.7) cm, and 26.3 (5.7) kg/m^2^, and 124 (54.4%) patients were obese. The median (interquartile range) of the average LAN measured by actigraph was 1.5 (0.5–4.5) lux, by photometer was 4.1(1.0–15.8) lux. LAN was divided into 4 categories from the lowest LAN to the highest LAN by quartile. The odds ratio (OR) for obesity was significantly higher in the group exposed to the highest lux than in the group exposed to the lowest lux (OR4.0, 95% confidence interval: 1.8–8.8, *p* < 0.001 by actigraph, OR2.6, 95% confidence interval: 1.2–5.6, *p* = 0.02 by photometer). In the multivariable logistic regression analysis, similar result was observed after adjusting for several confounding factors like age, gender, medications, and daily activity (OR3.5, 95% confidence interval: 1.5–8.1 by actigraph, OR2.3, 95% confidence interval: 1.0–5.3 by photometer). Furthermore, the patients with highest LAN lux had significantly higher body weight (adjusted mean, +10.1 kg; 95% confidence interval: 4.1–16.0, *p* = 0.001 by actigraph, +7.4 kg; 95% confidence interval: 1.3–13.4, *p* = 0.02 by photometer) and abdominal circumference (adjusted mean, +7.3 cm; 95% confidence interval: 1.9–12.6, *p* = 0.008 by actigraph, +6.2 cm; 95% confidence interval: 0.8–11.2, *p* = 0.03 by photometer) than those with lowest LAN lux. **Conclusions:** Significant association between light exposure at night and obesity was observed, and LAN exposure was also significantly associated with continuous data on body weight and abdominal circumference, independent of confounding factors. Further longitudinal investigations are necessary to validate the association between LAN exposure and obesity in schizophrenia.

## 5. Individual Differences in Light Response Predict Adjustment to Night Shift Work

**Authors**

Dr. Alisha Guyett-Australia-Flinders Health and Medical Research Institute: Sleep Health, Flinders University, Adelaide, Australia.

Dr. Ranjay Chakraborty-Australia-College of Nursing and Health Sciences, Flinders University, Adelaide, Australia.

Dr. Nicole Lovato-Australia-Flinders Health and Medical Research Institute: Sleep Health, Flinders University, Adelaide, Australia.

Dr. Jack Manners-Australia-Flinders Health and Medical Research Institute: Sleep Health, Flinders University, Adelaide, Australia.

Ms. Nicole Stuart-Australia-Flinders Health and Medical Research Institute: Sleep Health, Flinders University, Adelaide, Australia.

Dr. Duc Phuc Nguyen-Australia-Flinders Health and Medical Research Institute: Sleep Health, Flinders University, Adelaide, Australia.

Prof. Peter Catcheside-Australia-Flinders Health and Medical Research Institute: Sleep Health, Flinders University, Adelaide, Australia.

Dr. Hannah Scott-Australia-Flinders Health and Medical Research Institute: Sleep Health, Flinders University, Adelaide, Australia.

**Abstract**

**Background:** Individual variations in light sensitivity and daily circadian timing pose challenges for implementing effective lighting interventions seeking to delay circadian timing. This study tested predictors of individual responses to a circadian-informed lighting intervention during simulated night shift work to help better inform the design of potentially more effective personalisation of circadian lighting interventions. **Methods:** Nineteen healthy participants (12/7 male/female; mean ± SD aged 29 ± 10 yrs) underwent a post-illumination pupillary response (PIPR) test to assess retinal melanopic function (a measure of light sensitivity), and an 8-day lab study comparing two lighting conditions during simulated night shifts (00:00–08:00) with daytime sleep opportunities (10:00–19:00). Circadian changes were tracked through salivary melatonin sampling and continuous core body temperature monitoring via ingestible sensors. Regression analyses were used to examine predictors of dim light melatonin onset (DLMO) and core body temperature-minimum (Tmin) time changes from baseline- to post-lighting. Predictor variables in backward regression analyses included age, sex, measures of light sensitivity (6-sec and 30-sec PIPR), baseline circadian markers (Tmin and DLMO, tau and amplitude), and circadian light timing efficacy estimated from the daily Tmin position and total area under the circadian system phase response curve to light during the lighting period. **Results:** Backwards regression revealed six predictor variables: sex, 6-sec and 30-sec PIPR, baseline DLMO, circadian tau, and Tmin shift, accounted for 88% of the variance in DLMO shift (*p* = 0.007). 91% of the variance (*p* < 0.001) in the degree of Tmin shift was explained by two variables: baseline Tmin and circadian tau. **Conclusions:** Baseline circadian timing and retinal light sensitivity measures emerged as powerful predictors of adjustment to circadian-delaying lighting interventions. These findings support that more effective circadian adjustment can likely be achieved via objective physiological measures to help better personalise the timing of light interventions to treat circadian disruption.

**Keywords:** circadian rhythms; individual differences; light; light sensitivity

**Support:** This research is supported by the Commonwealth of Australia as represented by the Defence Science and Technology Group of the Department of Defence through the Research Network for Undersea Decision Superiority (research agreement number: 9334), with additional financial support from Flinders University and lighting provision support from REDARC.

## 6. The Life on Mars Protocol: Exploring the Impact of Clock Time Perception on Circadian Rhythms

**Authors**

Dr. Alisha Guyett-Australia-Sleep & Circadian Psychology Research Unit, Department of Psychology and Behavioural Sciences, Aarhus University, Aarhus, Denmark.

Dr. Cehao Yu-Denmark-Sleep & Circadian Psychology Research Unit, Department of Psychology and Behavioural Sciences, Aarhus University, Aarhus, Denmark.

Prof. Torben Sigsgaard-Denmark-Department of Public Health, Aarhus University, Aarhus, Denmark.

Prof. Kathryn Reid-United States-Center for Circadian and Sleep Medicine, Northwestern University, Chicago, USA.

Prof. Ben Colagiuri-Australia-School of Psychology, University of Sydney, Sydney, Australia.

Dr. Katharina Wulff-Sweden-Department of Molecular Biology, Umeå University, Umeå, Sweden.

Prof. Christian Cajochen-Switzerland-Centre for Chronobiology, Psychiatric University Hospital, University of Basel, Basel, Switzerland.

Dr. Lisa Wu-Denmark-Sleep & Circadian Psychology Research Unit, Department of Psychology and Behavioural Sciences, Aarhus University, Aarhus, Denmark.

Dr. Ali Amidi-Denmark-Sleep & Circadian Psychology Research Unit, Department of Psychology and Behavioural Sciences, Aarhus University, Aarhus, Denmark.

**Abstract**

**Background:** Circadian rhythms regulate multiple physiological and behavioural functions including sleep-wake cycles, hormone secretion, and cognitive performance. While light-dark cycles remain the primary zeitgeber for circadian entrainment, other environmental and internal cues may also influence circadian adjustment. Clock time dictates recurrent daily activities such as eating and sleep, which cues expectancies and may function as a potent conditioning stimulus. This abstract presents on the protocol used to explore the potential effect of time perception on the circadian system. Specifically, it investigates whether exposure to simulated clock time changes will result in shifts in circadian rhythm markers. We hypothesize that compared to a control group, exposure to simulated clock time changes will result in larger shifts in circadian markers. **Methods:** Forty healthy adults with normative sleep-wake patterns (aged 23–35) will be recruited for an experimental 5-day in-laboratory study. Participants will be randomised to either a simulated daily 1-h clock time delay condition using a fast running wall clock, or a no-delay control condition. To minimise participant bias, participants will be informed that the study examines the effect of circadian rhythms on a 25-h day broadly corresponding to the length of a day on Mars. Participants will be kept under a strict 16:8 light-dark cycle (100 lux, 2700 K). Daily repeated performance measures will include time production/estimation tasks conducted using a verbal estimation method, alongside cognitive tasks such as the psychomotor vigilance task and N-back task to assess vigilance, attention, and working memory. Circadian distal skin temperature and sleep/wake cycles will be recorded via actigraphy. Circadian phase of melatonin will be assessed via dim light melatonin onset on days 1, 3, and 5. **Discussion:** This abstract outlines a study that investigates how clock time perception might influence circadian rhythms. By investigating the potential role of clock time perception as a mechanism of circadian adjustment, the research aims to identify novel non-photic zeitgebers. These findings could provide insights into alternative strategies for circadian rhythm adjustment, particularly in contexts such as shift work where traditional light-based interventions may be limited or ineffective.

**Keywords:** time perception; time production; circadian rhythms; cognitive performance

**Funding:** This research is supported by the Independent Research Fund Denmark (10.46540/4256-00108B) with additional financial support from Aarhus University.

## 7. Impact of Circadian Adaptation on Sleep Architecture and Neurobehavioural Performance in Simulated Shiftwork

**Authors**

Dr. Shauni Omond-United States-Division of Sleep and Circadian Disorders, Departments of Medicine and Neurology, Brigham and Women’s Hospital; Division of Sleep Medicine, Harvard Medical School

Dr. Leilah Grant-United States-Division of Sleep and Circadian Disorders, Departments of Medicine and Neurology, Brigham and Women’s Hospital; Division of Sleep Medicine, Harvard Medical School

Dr. Melissa St. Hilaire-United States-Department of Computer and Data Sciences, Merrimack College

Dr. Laura Barger-United States-Division of Sleep and Circadian Disorders, Departments of Medicine and Neurology, Brigham and Women’s Hospital; Division of Sleep Medicine, Harvard Medical School

Dr. George Brainard-United States-Light Research Program, Department of Neurology, Thomas Jefferson University

Dr. Elizabeth Klerman-United States-Division of Sleep and Circadian Disorders, Departments of Medicine and Neurology, Brigham and Women’s Hospital; Division of Sleep Medicine, Harvard Medical School; Department of Neurology, Massachusetts General Hospital

Dr. Steven Lockley-United States-Division of Sleep and Circadian Disorders, Departments of Medicine and Neurology, Brigham and Women’s Hospital; Division of Sleep Medicine, Harvard Medical School

Dr. Shadab Rahman-United States-Division of Sleep and Circadian Disorders, Departments of Medicine and Neurology, Brigham and Women’s Hospital; Division of Sleep Medicine, Harvard Medical School

**Abstract**

**Introduction:** Shift workers commonly experience circadian misalignment, which disrupts sleep. We examined the impact of a dynamic lighting schedule on circadian adaptation, sleep architecture, and neurobehavioral performance in a simulated shiftwork paradigm. **Methods:** Healthy adults (*n* = 19, 10F, mean age [±SD] 36.2 ± 9.2 years) completed an 8-day inpatient protocol that included 4 consecutive simulated nightshifts during which the sleep-wake schedule was delayed 8 h relative to each participant’s habitual schedule. Participants were randomized to one of three dynamic lighting interventions during the four nightshifts that differed in illuminance, spectrum, and timing, and were expected to induce a phase delay. Dynamic LED lighting stimuli ranged from 25 to 700 melanopic equivalent daylight Illuminance (mEDI, or from 50 to 750 photopic lux). Circadian adaptation was assessed by comparing plasma dim light melatonin onset (DLMO) measured before and after the nightshifts. Sleep during an 8-h sleep opportunity was measured polysomnographically before the first nightshift (baseline) and after the fourth nightshift. The Karolinska Sleepiness Scale (KSS), Psychomotor Vigilance Task (PVT—auditory [AUD] and visual [V]), and a Probed Recall Memory (PRM) were conducted every 1–2 h throughout the wake episode. **Results:** The mean (±SEM) circadian phase-delay shift was −5.1 ± 0.3 h (range: −8.1 to −1.8 h) of the targeted 8-h. Sleep architecture did not differ across the three conditions, so data were combined for analyses. Compared to baseline, sleep after the fourth nightshift was associated with significantly reduced total sleep time (TST, 415.40 ± 20.24 vs. 377.2 ± 15.02 min, *p* < 0.01), stage 2 sleep (N2, 187.3 ± 13.16 vs. 151.7 ± 9.47 min, *p* < 0.01), and sleep efficiency (SlpEff, 86.05 ± 4.19 vs. 78.52 ± 3.11%, *p* < 0.01), and increased wake after sleep onset (WASO, 52.92 ± 19.15 vs. 84.79 ± 15.10 min, *p* < 0.05). Sleep onset latency (SOL) did not differ (18.02 ± 5.32 vs. 14.68 ± 2.45 min, *p* = 0.76). Greater circadian phase shifts, corresponding to more adaptation to the shifted sleep-wake schedule were correlated with increased TST (r = 0.70, *p* < 0.001), SlpEff (r = 0.69, *p* < 0.001), REM (r = 0.61, *p* < 0.01), and decreased WASO (r = −0.73, *p* < 0.001), but not SOL (r = 0.31, *p* = 0.19). There was no significant interaction between lighting condition and nightshift with respect to neurobehavioural performance, however lighting condition had a significant impact on KSS (*p* = 0.05) and nightshift had a significant impact on KSS, VPVT reaction time (RT), VPVT attentional failures, and AUDPVT RT (all *p* < 0.02). Individuals who had more wakeups throughout the sleep episode performed significantly worse on AUDPVT reaction time (r = 0.48, *p* = 0.04) and attentional failures (r = 0.48, *p* = 0.04), however, increased slow wave sleep was associated with more correct words in the PRM (r = 0.54, *p* = 0.01). Increased adaptation decreased reported sleepiness on the KSS (r = −0.55, *p* = 0.01), and increased the number of correct words scored in the PRM (r = 0.46, *p* = 0.04). **Conclusions:** The results show that greater circadian adaptation is associated with better sleep architecture and better scores on KSS and PRM. Additional studies are needed to optimize details of dynamic lighting schedules and lighting characteristics for preserving sleep quality and neurobehavioural performance in various shiftwork schedules.

## 8. The Impact of a Personalized Sleep Intervention on Shift Workers: Improved Subjective Sleep Without Objective Changes

**Authors**

Ms. Maaike van der Rhee-Netherlands-Department of Public Health, Erasmus University Medical Center, Rotterdam

Dr. Johanneke Oosterman-Netherlands-Department of Internal Medicine, Division Endocrinology, Erasmus University Medical Center, Rotterdam

Dr. Suzan Wopereis-Netherlands-Research Group Microbiology & Systems Biology, TNO, Netherlands Organization for Applied Scientific Research

Dr. Martijn Dolle-Netherlands-Centre for Health Protection, National Institute for Public Health and the Environment (RIVM)

Prof. Alex Burdorf-Netherlands-Department of Public Health, Erasmus University Medical Center, Rotterdam

Dr. Linda van Kerkhof-Netherlands-Centre for Health Protection, National Institute for Public Health and the Environment (RIVM)

Dr. Heidi Lammers-van der Holst-Netherlands-Department of Public Health, Erasmus University Medical Center, Rotterdam

**Abstract**

**Background:** Working during the night is associated with an increased risk for sleep problems and several chronic diseases. Night workers typically report shorter, less consolidated, and highly disrupted sleep during the day and increased sleepiness during night shifts. Interventions focused on sleep and nutrition can mitigate the short-term adverse effects of shift work under controlled conditions. However, in the field the adoption of a personalized approach may be a useful strategy to maximize these benefits. Therefore, the current study aims to evaluate the effects of a personalized sleep and nutritional advice on sleep duration and quality in real-life shift workers. **Methods:** This controlled intervention study included 57 healthy shift workers working 12-h shifts within the industry sector. Participants received tailored advice based on their individual-specific information such as baseline sleep outcomes, social obligations, and physiological markers. The sleep advice focused on sleep timing, multiple sleep episodes, and sleep hygiene education, while the nutritional guidance structured meal timing, macronutrient distribution, and energy intake. Sleep was assessed objectively through actigraphy at baseline, start intervention and post-intervention (averaging 13 night shifts, 6 early shifts and 20 free days per participant). Subjective sleep was measured with the Insomnia Severity Index at baseline, post-intervention and follow-up. Mixed-effects models with a random intercept were used, adjusted for age, chronotype and household. **Results:** Participants experienced on average shorter sleep duration when working night and early shifts, compared to recovery days. Neither intervention changed the objective sleep duration or quality. Participants who received the sleep advice had significantly higher insomnia scores at baseline, and their scores had decreased to match those of the other groups on post-intervention and during follow-up 8 months after the intervention. No such effects were observed for the nutritional guidance. **Conclusions:** Personalized sleep guidance improved subjective sleep experience (insomnia scores) but had no effect on objective sleep measures, highlighting the disconnect between subjective experience and objective sleep metrics.

**Keywords:** shift work; night shift; sleep; actigraphy; insomnia; personalized intervention

**Funding:** This study is funded by the project BioClock (with project number 1292.19.077) of the research program Dutch Research Agenda: Onderzoek op Routes door Consortia (NWA-ORC) which is (partly) financed by the Dutch Research Council (NOW) and the Dutch Ministry of Social Affairs and Employment (KV11.34).

## 9. Sleep Inertia Is a Biomarker of Circadian Misalignment and Drives the Association of Evening Chronotype with Psychiatric Disorders

**Authors**

Dr. Angus Burns-United States-Division of Sleep and Circadian Disorders, Departments of Medicine and Neurology, Brigham and Women’s Hospital; Division of Sleep Medicine, Harvard Medical School

Mr. Jakob Cherry-United States-Division of Sleep and Circadian Disorders, Departments of Medicine and Neurology, Brigham and Women’s Hospital

Dr. Stephanie Zellers-Finland-Institute for Molecular Medicine Finland, Helsinki Institute of Life Science, University of Helsinki, Helsinki, Finland

Dr. Daniel Windred-Australia-Flinders Health and Medical Research Institute: Sleep Health, Flinders University, Adelaide, Australia

Dr. Iyas Daghlas-United States-University of California, San Francisco

Dr. Nasa Sinnott-Armstrong-United States-Fred Hutchinson Cancer Research Foundation

Dr. Martin Rutter-United Kingdom-Division of Diabetes, Endocrinology and Gastroenterology, Faculty of Medicine, Biology and Health, University of Manchester

Dr. Christer Hublin-Finland-Working Ability and Working Careers, Finnish Institute of Occupational Health

Dr. Eleni Frigilikou-United States-Yale Department of Psychiatry

Dr. Renato Polimanti-United States-Yale Department of Psychiatry

Dr. Andrew Phillips-Australia-Flinders Health and Medical Research Institute: Sleep Health, Flinders University, Adelaide, Australia

Dr. Sean Cain-Australia-Flinders Health and Medical Research Institute: Sleep Health, Flinders University, Adelaide, Australia

Dr. Jaakko Kaprio-Finland-Institute for Molecular Medicine Finland, Helsinki Institute of Life Science, University of Helsinki, Helsinki, Finland

Dr. James Wyatt-United States-Rush University

Dr. Daniel Cohen-United States-Division of Neurology, Sentara Healthcare, Norfolk Virginia

Dr. Andrew McHill-United States-Oregon State Health University

Dr. Charles Czeisler-United States-Division of Sleep and Circadian Disorders, Departments of Medicine and Neurology, Brigham and Women’s Hospital; Division of Sleep Medicine, Harvard Medical School

Prof. Frank Scheer-United States-Division of Sleep and Circadian Disorders, Departments of Medicine and Neurology, Brigham and Women’s Hospital; Division of Sleep Medicine, Harvard Medical School

Dr. Hanna Ollila-United States-Broad Institute

Dr. Elizabeth Klerman-United States-Division of Sleep and Circadian Disorders, Departments of Medicine and Neurology, Brigham and Women’s Hospital; Division of Sleep Medicine, Harvard Medical School; Department of Neurology, Massachusetts General Hospital

Dr. Richa Saxena-United States-Division of Sleep and Circadian Disorders, Departments of Medicine and Neurology, Brigham and Women’s Hospital; Division of Sleep Medicine, Harvard Medical School; Department of Neurology, Massachusetts General Hospital

Dr. Jackie Lane-United States-Division of Sleep and Circadian Disorders, Departments of Medicine and Neurology, Brigham and Women’s Hospital

**Abstract**

**Background:** Despite the established link between circadian misalignment and many psychiatric disorders, clinically actionable biomarkers to identify at-risk individuals remain lacking. Evening chronotypes (“night-owls”) are disproportionately affected by psychiatric disorders, a disparity that may be attributed to circadian misalignment when external societal demands result in sleep timing that is early relative to internal circadian timing. This circadian misalignment heightens sleep inertia: the state of impaired alertness immediately after awakening. However, it is unknown whether subjective sleep inertia could be leveraged as a biomarker of circadian misalignment and therefore have clinical value as an intervenable mediator of risk for developing psychiatric disorders. **Methods & Results:** To assess this, we analyzed data from 54 individuals who lived in an inpatient, time-free environment for ~1-month under a circadian forced desynchrony protocol. The results revealed that there is a linear relationship between the magnitude of sleep inertia, measured by subjective sleepiness after awakening (KSS), and circadian misalignment, such that greater sleep inertia is associated with an increased probability of wake in the biological night. Our analysis of the longitudinal incidence of psychiatric disorders in the UK Biobank (*n* = 496,820) found that sleep inertia explained the relationship between evening chronotype and increased incidence of major depressive disorder, schizophrenia, generalized anxiety disorder and bipolar disorder. Evening chronotypes without sleep inertia were at no higher risk than morning chronotypes. Longitudinal analyses of suicide and depressed mood (CES-D score) in the Older Finnish Twin Cohort (*n* = 23,854) replicated this result. Twin and genome-wide association analyses of sleep inertia identified the trait to be heritable (Twin *H*^2^ = 0.40; SNP *h*^2^ = 0.08), be enriched for circadian rhythms genes, and share substantial genetic architecture with chronotype. Marginal and conditional Mendelian randomization analyses revealed that the causal effect of evening chronotype on these psychiatric disorders was driven by shared genetic architecture with sleep inertia. **Conclusions:** Collectively, these results challenge the notion that evening chronotype is a risk factor for psychiatric disorders *per se*, suggesting instead that evening types are at greater risk for psychiatric disorders due to circadian misalignment, for which sleep inertia acts as a biomarker. Sleep inertia may be an easily measurable and scalable biomarker to identify individuals at-risk of developing psychiatric disorders due to circadian misalignment.

## 10. Living in Biological Darkness III: Low-Level Morning Lighting Affects Depression Markers in Healthy Participants

**Authors**

Dr. Jan de Zeeuw-Germany-Charité–Universitätsmedizin Berlin, Institute of Physiology, Sleep Research & Clinical Chronobiology, Berlin, Germany

Dr. Claudia Nowozin-Germany-Charité–Universitätsmedizin Berlin, Institute of Physiology, Sleep Research & Clinical Chronobiology, Berlin, Germany

Mr. Martin Haberecht-Germany-St. Hedwig Hospital, Clinic for Sleep & Chronomedicine, Berlin, Germany

Mr. Sven Hädel-Germany-St. Hedwig Hospital, Clinic for Sleep & Chronomedicine, Berlin, Germany

Dr. Frederik Bes-Germany-Charité–Universitätsmedizin Berlin, Institute of Physiology, Sleep Research & Clinical Chronobiology, Berlin, Germany

Dr. Dieter Kunz-Germany-Charité–Universitätsmedizin Berlin, Institute of Physiology, Sleep Research & Clinical Chronobiology, Berlin, Germany

**Abstract**

**Background:** Affective disorders are associated with dysregulation of the hypothalamic-pituitary-adrenal (HPA) axis and certain changes in sleep patterns. The HPA and sleep-wake are circadian modulated and thus influenced by light exposure. Surprisingly, the impact of low-intensity daytime lighting—“*Living in Biological Darkness*”—on depressive tendencies seems underexplored. We recently reported that healthy people in urban environment were exposed to extremely low light levels during winter accompanied by a depression-like sleep pattern. This study aimed to examine how exposure to low light levels in the morning affects depression-related markers in healthy individuals. **Methods:** After keeping habitual bedtimes for a week, monitored through actigraphy, twenty healthy participants (mean ± SD: 24.5 ± 2.8 years; 10 females) were randomly assigned to receive either low intensity incandescent lighting (55 lx) or blue-enriched fluorescent lighting (800 lx, 3500 K) for five mornings (8:00–12:00) over a seven-day period. Assessments conducted before and after the intervention included two-night polysomnography, 32-h fractionated urine cortisol, salivary cortisol and melatonin levels, reaction time, and visual analogue scales for subjective daytime sleepiness and sadness. **Results:** Exposure to low-level incandescent lighting led to significantly higher urine cortisol levels in the evening (19:00–23:00; *p* = 0.021) and higher salivary cortisol in the afternoon (16:00; *p* = 0.026). Total sleep time was reduced by about 25 min after repetitive low-level lighting (*p* = 0.016) and slow-wave activity shifted from the second NREM-REM cycle to the third (*p* = 0.018). Additionally, subjective sleepiness (*p* = 0.023) and subjective sadness (*p* = 0.016) were significantly increased after low-level incandescent lighting. In contrast, blue-enriched lighting resulted in an increase in REM sleep towards the end of the sleep period. Circadian phase remained unchanged across both groups. **Conclusions:** Repeated exposure to low-intensity lighting in the morning was associated with increased cortisol levels and delayed slow-wave activity within nighttime sleep, changes previously observed in individuals with depressive disorders. These findings may provide insights into mechanisms underlying mood disturbances and may offer potential preventive strategies.

## 11. Post-Stroke Sleep and Circadian Disturbances: Experiences, Strategies, and Needs

**Authors**

Dr. Stephen Lau-Hong Kong-The Hong Kong Polytechnic University

Mr. Tyler Hood-United States-Washington University School of Medicine

**Abstract**

**Background**: Sleep and circadian disturbances are a common but often overlooked consequence of stroke, significantly affecting recovery, daily functioning, and the quality of life of stroke survivors. However, there is limited insight into the experiences and perspectives of stroke survivors facing sleep and circadian disturbances, which is crucial for developing targeted interventions to address these issues. **Objectives:** To explore the experiences, coping strategies, and needs of stroke survivors dealing with clinically significant sleep and circadian disturbances. **Methods:** We conducted semi-structured interviews with fifteen chronic stroke survivors living in the community who experienced sleep and circadian disturbances. The interviews were analyzed using thematic content analysis. **Results:** Nine key themes related to experiences emerged: (1) the impact of stroke on sleep, (2) factors worsening sleep, (3) factors improving sleep, (4) nature of sleep disturbances, (5) consequences of disrupted sleep, (6) perceptions of medication, (7) napping behaviors, (8) influence of sleep partners, and (9) help-seeking behaviors. Three themes on coping strategies emerged: (1) adaptive coping methods, (2) maladaptive coping methods, and (3) strategies recommended by healthcare providers. Three themes related to wishes and needs emerged: (1) desires for sleep interventions and increased knowledge about sleep and stroke, (2) communication and support within the healthcare system, and (3) usage of medications and equipment. **Conclusions:** This study underscores the complexity of sleep and circadian disturbances following stroke and emphasizes the need for individualized, multifaceted interventions to address the unique sleep challenges faced by stroke survivors. The findings also highlight the necessity for more attention and support from healthcare providers to incorporate sleep and circadian health into routine stroke care.

## 12. Daily Rhythmicity in Protein Levels and Atherosclerotic Cardiovascular Disease: An Epidemiological Cohort Study in UK Biobank

**Authors**

Dr. Raymond Noordam-Netherlands-Department of Clinical Epidemiology, Leiden University Medical Center

Prof. Ko Willems van Dijk-Netherlands-Department of Human Genetics, Leiden University Medical Center, Leiden

Dr. Diana van Heemst-Netherlands-Department of Internal Medicine, Section of Gerontology and Geriatrics, Leiden University Medical Center

Dr. Laura Kervezee-Netherlands-Group of Circadian Medicine, Department of Cell and Chemical Biology, Leiden University Medical Center

**Abstract**

**Background and aim**: It is widely acknowledged that daily variation in behavior, including timing of sleep, food intake and physical activity, can influence the risk of atherosclerotic cardiovascular disease. Many biological processes have a 24-h rhythm, which reflect different biological processes. However, it remains largely unknown to what extent variations in rhythmicity of protein levels are associated with the risk of incident atherosclerotic cardiovascular disease. Here, we aimed to investigate the associations between 24-h variation in protein levels and incident coronary artery disease in a large population-based cohort study of middle-aged and older people. **Methods**: The present study was performed using data collected in UK Biobank, in a cohort of participants (aged between 40 and 70 years at inclusion) free of a history of coronary artery disease. During the baseline examination, non-fasting blood was collected from participants at a random time point between 8 AM and 8 PM. In these blood samples, levels of 2923 proteins were measured using OLINK proteomics. Linearized cosinor analyses with a fixed period of 24 h were performed to identify proteins with evidence of a daily rhythm, followed by prospective cox-proportional hazard models to study the association between variation in 24-h rhythms of the protein levels with incident coronary artery disease. The statistical significance of the 24-h variation in protein levels was assessed using Wald tests to compare the cosinor model to a null model without cosinor (interaction) coefficients. **Results**: In a sample free of atherosclerotic cardiovascular disease at baseline (Nmax = 48,967, 55% women, mean [standard deviation] age of 56.4 [8.1] years), and after accounting for multiple testing using the false-discovery rate, a total of 2246 protein levels showed evidence for a 24-h rhythm (*p* < 0.05). Of these, there was only evidence that variation in rhythmicity of SPON2 levels was associated with the risk of coronary artery disease. **Conclusions**: Within the present large study sample, the vast majority of the examined proteins showed evidence for a 24-h rhythm in the circulation. However, with the exception of SPON2, variation in 24-h protein rhythmicity seems not to be associated with incident atherosclerotic cardiovascular disease likely suggesting that daily rhythmicity in protein levels is an unlikely target for cardiovascular disease prevention.

**Keywords:** proteomics; circadian rhythm; cardiovascular disease

**Funding:** The present work was supported by a grant from the Dutch Research Council (NWO, Dutch National Research Agenda, Research along routes by consortia, 2021–2026, BioClock: the circadian clock in modern society; to Diana van Heemst and Laura Kervezee).

## 13. A Naturalistic Light Monitoring Study Identifies Correlations Between Acute Light Exposure and Positive Mood

**Authors**

Ms. Chloe Roddis-United Kingdom-University of Manchester

Dr. Nina Milosavljevic-United Kingdom-University of Manchester

Dr. Beatriz Bano Otalora-United Kingdom-University of Manchester

Dr. Altug Didikoglu-Türkiye-Izmir Institute of Technology

Dr. Catherine Harmer-United Kingdom-University of Oxford

Dr. Amy Gillespie-United Kingdom-University of Oxford

**Abstract**

**Background:** Light plays a vital role beyond visual perception, influencing mood, cognition, and physiological processes. As a non-invasive and accessible therapeutic tool, light exposure has the potential to enhance sleep, mood, and overall well-being. However, to effectively harness these benefits, a deeper understanding of how naturalistic light exposure impacts emotional states in real-world contexts is needed. This study aimed to investigate the relationship between recent and cumulative light exposure and subjective mood in everyday life. **Methods:** Fifty-one healthy adults participated in a one-week observational field study, during which they wore light sensors (Condor Actlumus) to measure melanopic Equivalent Daylight Illuminance (Melanopic-EDI). Participants also wore Fitbit devices to track sleep and physiological activity. Each day, participants completed online surveys, including sleep diaries and subjective mood assessments. Mood ratings were collected multiple times daily on visual analogue scales (0–100) for seven mood variables: Happy, Relaxed, Energetic, Motivated, Carefree, Enjoyment, and Accompanied. Analyses focused on both acute light exposure (from the preceding 30 min up to 4 h) and daily cumulative exposure above melanopic-EDI thresholds (time above thresholds: 1000 and 250 melanopic-EDI). **Results:** Initial analyses showed individual variation in the relationship between light exposure and mood ratings, with positive associations observed for all mood variables at 30-min intervals. Further investigation using linear mixed models identified consistent positive relationships between light exposure and specific mood states. Energetic, motivated and happiness mood scores were positively associated with light exposure over intervals ranging from 30 min to 4 h, with the strongest associations typically observed at 30 min. Sustained daily exposure to higher light levels, measured by time spent above 1000 and 250 melanopic lux, was also associated with higher average ratings of happiness, relaxation, and enjoyment. After adjusting for covariates, such as time awake and subjective morning sleepiness (measured by the Karolinska Sleepiness Scale), the strength of these associations was reduced, however the effects remained significant at 3 to 4 h. This suggests that some of the observed effects may be influenced by shared temporal or sleep-related factors but also that the effect of direct light is evident on these mood measures. It also indicates that different mechanisms may underlie the acute effects of light exposure on energy and motivation compared to its effects on other subjective mood measures. **Conclusions:** These findings highlight both acute hourly and sustained daily associations between naturalistic light exposure and positive mood in daily life, with variation in effects depending on the mood variable and time interval considered. Exposure to higher melanopic-EDI was most consistently associated with feelings of energy, motivation and happiness in the short term, while cumulative light exposure across the day related to more general positive affect, including happiness, relaxation, and enjoyment. The study underscores the importance of considering both melanopic-EDI, timing and duration when investigating light’s impact on mood. Further research will explore underlying mechanisms with a potential to inform intervention strategies aimed at optimising light exposure for mental health and well-being.

**Keywords:** light exposure; mood; melanopic illuminance; circadian health; well-being; naturalistic study; wearable sensors

**Funding:** This research was supported by a BBSRC Doctoral Training Partnership (DTP) CASE studentship in partnership with Signify.

## 14. Personalized Multimodal Circadian Intervention Study in Older Adults with Poor Sleep Quality

**Authors**

Mr. Gunnar Deuring-Switzerland-Centre for Chronobiology, Psychiatric University Hospital, University of Basel, Basel, Switzerland.

Ms. Sophie Reckels-Switzerland-Centre for Chronobiology, Psychiatric University Hospital, University of Basel, Basel, Switzerland.

Ms. Johanna Otte-Switzerland-Centre for Chronobiology, Psychiatric University Hospital, University of Basel, Basel, Switzerland.

Dr. Christian Epple-Switzerland-Centre for Chronobiology, Psychiatric University Hospital, University of Basel, Basel, Switzerland.

Dr. Benedikt Reuthebuch-Switzerland-Centre for Chronobiology, Psychiatric University Hospital, University of Basel, Basel, Switzerland.

Dr. Corrado Garbazza-Switzerland-Centre for Chronobiology, Psychiatric University Hospital, University of Basel, Basel, Switzerland.

Dr. Martin Meyer-Switzerland-Psychiatric Hospital of the University of Basel, Basel, Switzerland.

Dr. Helen Slawik-Switzerland-Psychiatric Hospital of the University of Basel, Basel, Switzerland.

Prof. Christian Cajochen-Switzerland-Centre for Chronobiology, Psychiatric University Hospital, University of Basel, Basel, Switzerland.

Prof. Anne Eckert-Switzerland-Psychiatric Hospital of the University of Basel, Basel, Switzerland.

Prof. Aki Kawasaki-Switzerland-University of Lausanne, Switzerland.

Prof. Susanne Jaeggi-United States-Northeastern University, Boston, USA.

Dr. Mirjam Münch-Switzerland-Centre for Chronobiology, Psychiatric University Hospital, University of Basel, Basel, Switzerland.

**Abstract**

**(1) Background:** Circadian interventions in different populations typically focus on a single target strategy to improve zeitgeber strength, i.e., by applying timed light exposure or physical activity to improve sleep-wake rhythms. Our approach was to use a multimodal personalized circadian intervention with a co-design approach over six months to improve subjective sleep quality in older adults with sleep complaints. **(2) Methods:** The study was designed as a semi-blinded, randomised controlled trial and included 66 older adults > 65 years (mean age ± SD: 72.2 ± 5.7; 40 females, 26 male). The inclusion criteria for sleep complaints was a global score > 5 on the Pittsburgh Sleep Quality Index (PSQI). Participants were randomly assigned to either the intervention (IG) or the control group (CG). After one baseline week (BL), during which measures were collected at home, the IG received low-threshold personalized instructions regarding sleep, light exposure, physical activity, and meal times. These instructions were based on BL measures and adjusted at the first home visit upon discussion with participants, and then again during the home visit after three months to be continued for another three months. The CG underwent identical procedures but received general sleep hygiene recommendations. Home measures by the participants were again performed for two weeks prior to the second and third visit. These included rest-activity recordings (Motion Watch8, Camntech UK), continuously worn light sensors, saliva collection for DLMO assessments, and questionnaires. Linear mixed models (adjusted for age and sex) were applied to determine whether the PSQI improved for the IG over six months, as compared to the CG. **(3) Results** At BL, the PSQI was on average 10.5 (=estimated marginal mean, EMM; Confidence Interval; CI: 9.8–11.2). It significantly improved after three months (EEM = 8.2; CI: 7.4–9.1; *p* < 0.001), and further improved after six months as compared to BL (EEM = 7.5; CI: 6.7–8.4; main effect of time; *p* < 0.05), with medium to large effect sizes (Cohen’s d: 0.36–1.32). Age only marginally impacted the results, but women reported significantly lower sleep quality in the PSQI overall than men. There were, however, no significant interaction effects of both groups with measurement time or with sex on the PSQI, indicating, that both, the IG and the CG experienced similar benefits in terms of better subjective sleep quality. **(4) Conclusions** Subjective sleep quality improved similarly in both groups, indicating the effectiveness of the personalised multimodal approach, but also of the general sleep hygiene recommendations. These findings highlight the fact that sleep quality can be improved even with low threshold recommendations, especially when applied over a longer period of time (i.e., several months).

**Keywords:** aging; sleep; daylight; time restricted eating; physical activity

**Funding:** This project is financially supported by the VELUX Stiftung (Switzerland) as part of the international integrative Circadian Human Daylight Platform (iHCDP; www.ihcdp.org).

## 15. Gestational Circadian Rhythm Disruption by a “Short Day” Light Cycle Engenders Autistic-like Behavioral Changes in the Offspring Exacerbated by High-Fat Diet

**Authors**

Mr. Ismaheel Adeniyi-United States-Rutgers University

Dr. Michael Oraebosi-United States-Rutgers University

Dr. Ruifeng Cao-United States-Rutgers University

**Abstract**

**Background:** Early-life adversity increases the risk of neurological and psychiatric disorders in later life, For example, prenatal and early postnatal stressors shape physiological and behavioral traits in the adulthood. The body’s circadian rhythms are naturally regulated by the environmental light/dark cycles, to synchronize the internal physiological and metabolic processes with the external environment. Epidemiological evidence supports that disruption of the body’s circadian rhythms, as seen in shift work, light at night, and insomnia, etc, may increase the risk of mental disorders. The transgenerational effects of circadian disruption on offspring mental health, however, remain to be fully understood. Here, we report a novel mouse model of gestational circadian disruption, the “short day” mouse, and its neuropathological and behavioral changes reminiscent of autistic-like traits, highlighting a significant role for environmental light cycle on neurodevelopment. **Methods:** Adult (8–12 week old) C57BL/6J breeders were maintained on a 12 h/12 h light/dark (LD) cycle, and shifted to an 8 h/8 h short day (SD) cycle immediately after mating. Mated females were separated from males and kept in the SD cycle until they gave birth, when the dam and the offspring were returned to the 12 h/12 h cycle for the rest of the experiment. Control (CTR) mice were bred and kept in the 12 h/12 h LD cycle constantly. The CTR and SD offspring mice were subject to a battery of behavioral tests at 8~12 weeks of age, when they are on normal chow. In a seperate cohort, postnatal 12 to 29 weeks, CTR and SD offspring mice were provided high fat diet (60 kcal% Fat) ad lib from 12 to 29 weeks and tested at 20–29 weeks of age. Thus, the study has mice in four groups: controls on normal diet (CTRND), CTR on the high-fat diet (CTRHFD), SD on normal diet (SDND) and SD on the high-fat diet (SDHFD). Mice were evaluated for behavioral changes related to anxiety, depression, cognition, repetitive behavior, and sociability. After behavioral tests, mice were sacrificed for brain immunohistochemistry to detect the distribution of the following proteins in the brain: phospho-S6, phospho-eIF4E, Iba1, and NeuN. **Results:** Anxiety- and depressive-like behaviors were assessed using the open field test (OFT), elevated plus maze (EPM), and forced swim test (FST). SDND mice exhibited significantly more anxiety- and depressive-like behaviors than CTRSD mice, as shown by reduced time in the center in the OFT, open arm in the EPM, and increased immobility in FST. These behavioral changes were worsened in SDHFD mice. In Novel object recognition (NOR) test, SDND ehited impaired recognition memory and it was not affected by HFD. However, in object location memory (OLM) test, the spatial memory deficits in SDND mice were exacerbated by HFD. Mouse repetitive behaviors were assessed using the marble burying and grooming tests. We found increased marble burying and self-grooming in SDND mice, both further increased in SDHFD mice, indicating that HFD exacerbates repetitive behaviors in SD mice. We used the three-chamber test to assess mouse sociality. We found impaired sociability and preference for social novelty in SDND mice compared to CTRND mice, which were also exacerbated by HFD. By immunostaining, we found increased levels of phospho-S6 and phospho-eIF4E in the mouse hippocampus of SDND and SDHFD mice, indicating hyperactivation of the mTORC1 and the MAPK pathways. Further, we found increased level of Iba1 expression in these mice, indicating significant microglia activation in the SDND and SDHFD mice. The expression of NeuN remains unchanged, indicating normal neuronal architecture in the hippocampus, hypothalamus, and prefrontal cortex. **Conclusions:** The current study demonstrates that gestational circadian disruption by environment light can lead to long-lasting behavioral and neurochemical changes in the adult offspring, which can be exacerbated by unhealthy diet in the adulthood. The findings support a concerted role of prenatal circadian disturbance and dietary style in sculpting the adult brain and behavior.

## 16. Alertness Optimization for Shift Workers Using a Physiology-Based Mathematical Model

**Authors**

Mr. Zidi Tao-United States-Department of Electrical, Computer, and Systems Engineering, Rensselaer Polytechnic Institute

Prof. Agung Julius-United States-Department of Electrical, Computer, and Systems Engineering, Rensselaer Polytechnic Institute

Prof. Wen John-United States-Department of Electrical, Computer, and Systems Engineering, Rensselaer Polytechnic Institute

**Abstract**

Sleep is vital for maintaining cognitive function, facilitating metabolic waste removal, and supporting memory consolidation. However, modern societal demands, particularly shift work, often disrupt natural sleep patterns. This disruption can induce excessive sleepiness among shift workers in critical sectors such as healthcare and transportation and increase the risk of accidents. The main factors contributing to this issue are misalignments of circadian rhythms and enforced sleep-wake schedules. Adjusting circadian rhythms can be regarded as a control problem with inputs in the form of light and sleep schedules. In this presentation, we address the model-based alertness optimization problem by optimizing light and sleep schedules to improve the cognitive performance of shift workers. The three-process model, which is phenomenology-based, has previously been used in the alertness optimization problem to predict the alertness of shift workers. However, a newer physiology-based model has demonstrated greater accuracy in predicting alertness than the phenomenology-based model. The dynamics of the physiology-based model involves stiff differential equations with widely varying time scales. This poses a challenge in optimization because of the high computational cost and the potential numerical instability of running simulations of the system. To overcome the challenge, we use singular perturbation techniques to reduce the dynamics of the system to a nonstiff, differentiable dynamics. This reformulation facilitates the application of the calculus of variation and the gradient descent method to find the optimal light and sleep schedules that maximize subjective alertness of shift workers. Optimal sleep and lighting schedules can be effectively solved with a gradient-based solver. Our approach is validated through numerical simulations that demonstrate that the proposed model’s prediction of shift workers’ alertness matches experimental data. Simulations of optimal sleep and lighting schedules indicate that late naps can improve alertness in shift workers. However, a more effective approach is to shift the circadian clock with a pre-optimized light and sleep schedule to maximize alertness during shift work. That is, when optimizing alertness during waking hours over multiple-day work shifts, the optimal schedules delay the circadian clock of shift workers to align with nighttime activity. This work was supported by the Army Research Office through Grants W911NF-22-10039 and by the National Science Foundation (NSF) through Grant DMS-2037357.

## 17. Timekeeping in the Brain: How Circadian Clocks and the Environment Shape Mood

**Author**

Dr. Diego Fernandez-United States-Cincinnati Children’s Hospital

**Abstract**

Mood disorders, including major depressive disorder and bipolar disorder, affect millions worldwide, yet their underlying biological mechanisms remain elusive. While genetics, environment, and lifestyle all contribute to mental health, emerging evidence suggests that dysregulation of clock gene expression plays a pivotal role. Clock genes regulate the mammalian central pacemaker within the suprachiasmatic nucleus (SCN) of the hypothalamus, which synchronizes rhythmic physiological and behavioral processes. However, circadian timekeeping extends beyond the SCN, influencing mood-related brain regions, including cortical, thalamic, and limbic structures. Recent analyses of postmortem brain tissue from individuals with major depression have revealed significant dysregulation of both clock genes and hundreds of clock-controlled genes across the limbic system. This raises a fundamental question: Is disrupted clock gene expression a cause of mood disorders, or merely a consequence? To address this, our research investigates whether environmental stressors, such as irregular light exposure, induce circadian disturbances that trigger mood deficits. We have found that mood-regulating brain circuits, particularly those involved in light processing, exhibit disrupted clock gene expression in mice exposed to irregular light rhythms. By examining the interplay between light-sensitive brain circuits and circadian gene expression, we aim to uncover how these mechanisms influence mood regulation and psychiatric disorders. Understanding these pathways may provide crucial insights into the role of circadian disruption in mental illness, paving the way for novel therapeutic interventions.

## 18. PerfectFit@Night: An Intervention to Enhance Sleep, Fatigue, and Recovery for Healthcare Shift Workers

**Authors**

Dr. Heidi Lammers-van der Holst-Netherlands-Department of Public Health, Erasmus University Medical Center, Rotterdam, The Netherlands

Ms. Fleur van Elk-Netherlands-Department of Public Health, Erasmus University Medical Center, Rotterdam

Dr. Suzan Robroek-Netherlands-Department of Public Health, Erasmus University Medical Center, Rotterdam

Prof. Alex Burdorf-Netherlands-Department of Public Health, Erasmus University Medical Center, Rotterdam

Dr. Karen Oude Hengel-Netherlands-Netherlands Organization for Applied Scientific Research TNO, Leiden

**Abstract**

**Background:** Night work, crucial for 24/7 healthcare, has negative health impacts. Targeted workplace health promotion is highly needed, therefore we developed a multi-faceted ‘PerfectFit@Night’ intervention to improve sleep, recovery and fatigue among shift workers in healthcare. The aim of this study is to evaluate the effects and implementation of PerfectFit@Night using the RE-AIM model, designed to assess Reach, Effectiveness, Adoption, Implementation and Maintenance of the intervention. **Methods:** A 3-months intervention with a prospective pre–post study design was implemented, including two baseline measurements and two follow-up measurements after 3 and 6 months (*n* = 210) after the intervention. PerfectFit@Night consisted of both environmental (provision of a powernap bed and healthy food at night; a workshop ‘healthy’ rostering) and individual components (e-learning and sleep coaching). A compiled survey of standardized questionnaires was sent around to shift workers at twelve departments of the Erasmus University Medical Center in The Netherlands to measure night shift related insomnia, overall sleep quality, sleep duration after night shifts, need for recovery and fatigue. Effectiveness was analyzed using mixed effects models. Reach, Adoption, Implementation and Maintenance were evaluated using the first follow-up data on usage and satisfaction, supplemented with qualitative data from interviews and logbooks. **Results:** The PerfectFit@Night intervention significantly reduced night shift-related insomnia (−11%-points, 95% CI: −19%, −4% at three months), need for recovery (β: −2.45, 95% CI: −4.86, −0.03 at six months) and fatigue (OR: 0.46, 95% CI: 0.25, 0.86 at six months). No changes were found for subjective sleep quality and sleep duration. These results were attributed to the combination of intervention elements rather than any specific element alone. Implementation barriers and facilitators were identified for each intervention component at individual (e.g., dietary preferences), organizational (e.g., work responsibilities), and workplace levels (e.g., location of the power nap bed), as well as for the intervention itself (e.g., useful content in e-learning). While satisfaction was high and a preference for continuation was expressed, the integration of the intervention into daily routines was limited. Facilitators for future implementation include a positive attitude toward the intervention, clear guidelines for its components, appointing shift workers as ambassadors, and creating suitable conditions in terms of work demands and the intervention components. **Conclusions:** The multi-faceted PerfectFit@Night intervention showed a positive effect on sleep, fatigue and recovery. The RE-AIM approach, combining both effect and implementation evaluation, identified important barriers and facilitators to improve future implementation of workplace health promotion programs for night shift workers.

## 19. Ethnic Differences in Pupillary Light Response Mediated by Intrinsically Photosensitive Retinal Ganglion Cells

**Authors**

Prof. Shigekazu Higuchi-Japan-Department of Human Life Design and Science, Faculty of Design, Kyushu University

Mr. Kazuki Imaizumi-Japan-Graduate School of Design, Kyushu University

Mr. Yusuke Nakazawa-Japan-Graduate School of Design, Kyushu University

Ms. Nuo Xu-Japan-Graduate School of Design, Kyushu University

Dr. Taisuke Eto-Japan-Department of Ophthalmology, Keio University School of Medicine

Dr. Toshiyuki Hayakawa-Japan-Faculty of Arts and Science, Kyushu University

Prof. Sei-ichi Tsujimura-Japan-Faculty of Design and Architecture, Nagoya City University

**Abstract**

**Background:** With the widespread use of artificial lighting and electronic devices, exposure to light at night has increased, raising concerns about its adverse effects on circadian rhythms and sleep. Although ethnic differences in light sensitivity are known to exist, they have not been thoroughly investigated. Intrinsically photosensitive retinal ganglion cells (ipRGCs) play a key role in mediating the non-image-forming effects of light. We assessed ethnic differences in the non-visual effects of light between East Asian and European populations by measuring steady-state pupillary responses to metameric light stimuli under varying illuminance and color temperature conditions. **Methods:** Participants included 18 East Asian adults (mean age 22.4 ± 1.5 years, 9 females) and 17 European adults (mean age 25.47 ± 3.81 years, 10 females), all with normal color vision and no ocular diseases. Light conditions combined two correlated color temperatures (3000 K and 6000 K) and three vertical illuminance levels at eye level (100, 400, and 1100 lx). For each condition, metameric stimuli were designed to selectively modulate ipRGC stimulation while maintaining S-, M-, and L-cone excitation constant. Pupil diameter was measured for 90 s during each light stimulus period. The pupillary constriction rate was calculated based on the pupil diameter before each light stimulus. **Results:** Significant main effects for all factors included ethnicity, illuminance, color temperature, and ipRGC stimulation level. The European group showed significantly greater pupillary constriction than the East Asian group. Higher ipRGC stimulation was associated with greater constriction. Dose-response curves using melanopic equivalent daylight illuminance (mEDI) as the independent variable revealed a significantly higher 50% effective dose (ED_50_) in the East Asian group than in the European group. **Conclusions:** This study assessed ipRGC reactivity via pupillary light response and found that the European group was more sensitive to light than the East Asian group.

## 20. The Effect of Exercise Timing on Insomnia and Sleep Quality: Evidence from the ON TIME Study

**Authors**

Ms. Gali Albalak-Netherlands-Department of Internal Medicine, Section of Gerontology and Geriatrics, Leiden University Medical Center

Dr. Raymond Noordam-Netherlands-Department of Clinical Epidemiology, Leiden University Medical Center

Mrs. Marjan van der Elst-Netherlands-Department of Internal Medicine, Section of Gerontology and Geriatrics, Leiden University Medical Center

Mr. Thomas Drop-Netherlands-Department of Internal Medicine, Section of Gerontology and Geriatrics, Leiden University Medical Center

Mr. Eloy Caneda Cabrera-Netherlands-Department of Internal Medicine, Section of Gerontology and Geriatrics, Leiden University Medical Center

Mr. Luuk Oudendijk-Netherlands-Department of Internal Medicine, Section of Gerontology and Geriatrics, Leiden University Medical Center

Dr. Marijke Gordijn-Netherlands-Chrono@Work & Chronobiology Unit, Groningen Institute for Evolutionary Life Sciences, University of Groningen

Dr. Laura Kervezee-Netherlands-Group of Circadian Medicine, Department of Cell and Chemical Biology, Leiden University Medical Center

Dr. Vasileios Exadaktylos-Netherlands-Center for Human Drug Research, Leiden

Prof. David van Bodegom-Netherlands-Leyden Academy on Vitality and Ageing

Dr. Diana van Heemst-Netherlands-Department of Internal Medicine, Section of Gerontology and Geriatrics, Leiden University Medical Center

**Abstract**

**Background** Insomnia is a common condition in older people. Sufficient physical activity is known to improve sleep quality. Despite the mounting evidence from epidemiological/observational studies about physical activity timing and health, the effect of timing of physical activity on insomnia and sleep quality has yet to be determined. We aimed to study the effect of timed exercise regimens (morning and evening) on insomnia severity, the circadian system, and objective and subjective sleep quality parameters in older people with insomnia. **Methods** Most important inclusion criteria were: (1) aged 60–80 years, (2) retired, (3) (sub)clinical insomnia (score ≥ 10 measured by the Insomnia Severity Index, ISI). The effect of structured and coached physical activity in the morning (10:00–11:00) compared to evening (19:30–20:30) was studied using a randomized cross-over design of 14 days per intervention. Insomnia severity was measured at the end of each intervention with the ISI. Sleep quality parameters, including duration, latency, efficiency, sleep stages and, midpoint of sleep were assessed using the Withings Sleep Analyzer (Withings France SA, Issyles Moulineaux, France) throughout the interventions. In addition, two questions about subjective sleep quality were asked with a smart phone app every morning. **Results** 36 overall healthy older adults with (sub)clinical insomnia (mean ISI score 14.3 ± 3.3) were included in the study of whom 27 completed the study (mean age 69.8 ± 5 years, 63% women). Compared to baseline, ISI scores decreased after both the active morning (delta: −2.5 point; 95%CI 1.14, 3.83; *p* ≤ 0.001) as well as the active evening intervention (delta: −2.0 point; 95%CI 0.63, 3.38; *p* = 0.006) compared to baseline ISI. We observed in the sleep-wake rhythm that the midpoint of sleep was significantly earlier during the active morning intervention to compared to the active evening intervention (03:40 vs. 04:00; delta: 20 min; 95%CI −31, −8; *p* = 0.001). There were no clinical nor statistical significant differences in the subjective sleep quality outcomes between the active morning and active evening period in the total population. **Conclusions** We did not observe a significant effect of physical activity timing on changes in most examined sleep quality parameters, possibly due to the relatively small sample size. Interestingly, we do find that morning physical activity results in an earlier midpoint of sleep-wake rhythm, which has been associated with better health in older adults with sleep problems. We emphasize the need of intervention studies with large study populations to support the conceptualization of optimal circadian timing of physical activity to promote better sleep and health.

**Keywords:** insomnia; sleep quality; physical activity timing; exercise; circadian rhythm

**Funding:** The present work was supported by a grant from the Dutch Research Council (NWO, Dutch National Research Agenda, Research along routes by consortia, 2021–2026, BioClock: the circadian clock in modern society (project number: 1292.19.077)); to Diana van Heemst, David van Bodegom, and Laura Kervezee.

## 21. Sensing Light for Circadian Regulation

**Author**

Prof. Michael Do-United States-Boston Children’s Hospital Harvard Medical School

**Abstract**

Mammals sense light for sight as well as for subconscious processes that include the regulation of circadian rhythms, sleep, and mood. These visual functions are initiated by photoreceptive cells that are diverse in form, varied in function, divergent in the downstream circuits they drive, and conserved to different degrees across species. This seminar concerns how photoreceptive mechanisms are tailored to their tasks. It will focus on circadian photoregulation, discuss specializations at several scales of biological organization, and draw comparisons between the nocturnal rodent and diurnal primate.

## 22. Effects of Sleep Deprivation, Recovery, and Time-of-Day on the Astrocyte Proteome

**Authors**

Prof. Ashley Ingiosi-United States-The Ohio State University

Ms. Caroline Jipa-United States-The Ohio State University

Mr. Andrew Brown-United States-The Ohio State University

Dr. Guihua Yue-United States-Washington State University

Dr. Christine Muheim-United States-Washington State University

Ms. Kaitlyn Ford-United States-Washington State University

Dr. Bhagwat Prasad-United States-Washington State University

Dr. Lucia Peixoto-United States-Washington State University

Dr. Marcos Frank-United States-Washington State University

**Abstract**

**Background:** Sleep is regulated by circadian and homeostatic processes. Though circadian regulatory elements are comparatively well understood, the cellular and molecular mechanisms that homeostatically balance sleep and wakefulness are less defined. We recently showed astrocytes respond dynamically to sleep, wake, and sleep loss, and disrupting astrocyte intracellular signaling impairs the brain’s ability to encode and accumulate sleep need. To better define the relationship between astrocyte signaling and sleep homeostasis, we conducted proteomic analyses of cortical astrocytes from rested, sleep deprived, and recovered mice. **Methods:** Male mice (8-weeks-old) were split into sleep deprived (SD; *n* = 33) and homecage control (HC; *n* = 33) groups. SD mice were sleep deprived for 6 h starting at light onset (i.e., Zeitgeber time (ZT) 0) using gentle handling and then either sacrificed immediately (SD6, *n* = 18) without opportunity for sleep or after 3 h of recovery sleep (RS3, *n* = 15) and brains collected. HC mice were left undisturbed but similarly sacrificed at ZT6 (HC6; *n* = 18) or ZT9 (HC9; *n* = 15) to serve as time-of-day controls. Samples were pooled—producing 5–6 biological replicates per condition—and cortical astrocytes were isolated from each pooled sample using a magnetic bead-based approach. Samples were then subjected to untargeted ultra performance liquid chromatography tandem mass spectrometry to quantify astroglial protein expression. We used removal of unwanted variation (RUV) analysis (k = 7) to normalize data and identify proteins differentially expressed between groups (adj. *p*-value = 0.05). Pathway analysis was then used to group differentially expressed proteins (DEPs) by function using gene ontology terms from NIH’s DAVID database. **Results:** Mass spectrometry detected 6011 total astroglial proteins across all samples. We filtered this dataset to 3276 proteins that were detected in at least 21 of the 22 total samples with a minimum intensity threshold of 50,000. Only 74 proteins were differentially expressed immediately after sleep deprivation (i.e., SD6) relative to HC6. These proteins were mostly upregulated and associated with chromatin structure. Downregulated proteins were associated with endocytosis. After recovery sleep (i.e., RS3), however, we identified 167 and 785 DEPs compared to HC9 and SD6, respectively. We found that recovery sleep upregulated proteins involved in mRNA processing and DNA binding, but downregulated proteins involved in cell structure, trafficking, and metabolism. By contrast, time-of-day upregulated metabolic-associated proteins at ZT9 (i.e., HC9) compared to ZT6 (i.e., HC6), and proteins associated with translation were downregulated as determined by 252 identified DEPs. **Conclusions:** We showed recovery from sleep deprivation has a greater impact on the astroglial proteome than sleep deprivation itself. This finding suggests astrocytes contribute to the homeostatic processes that rebalance sleep and wakefulness, and astroglial proteins associated with transcription, trafficking, cell structure, and metabolism are associated with this homeostatic recovery. Circadian processes impact astroglial metabolism as well, but in an opposite direction from homeostatic processes. Now, we are defining a role for the astroglial phosphoproteome in these homeostatic and circadian processes, and we identified high-confidence candidate proteins to further uncover a mechanistic role for astrocytes in sleep homeostasis.

## 23. Learning to Sleep: Evaluating the Impact of a Sleep and Circadian Rhythms Course on College Student Sleep Health

**Authors**

Prof. J. Roxanne Prichard-United States-University of St. Thomas

Mr. Abdul Abdullahi-United States-University of St. Thomas

Mr. Matthew Gehl-United States-University of St. Thomas

Mr. John McGill-United States-University of St. Thomas

Ms. Arianna Sanchez-United States-University of St. Thomas

Ms. Holly Tekle-United States-University of St. thomas

**Abstract**

**Introduction:** College students frequently experience insufficient and irregular sleep due to factors such as late-night studying, academic stress, evening light exposure, caffeine consumption, work responsibilities, and social activities. Poor sleep has been linked to heightened rates of anxiety and depression, and students who maintain regular, adequate sleep schedules tend to perform better academically. As such, interventions that raise awareness about sleep and circadian health may lead to improvements in students’ mental, physical, and academic well-being. This study investigates whether participation in a semester-long course on Sleep and Circadian Rhythms, which includes self-monitoring of sleep patterns, can serve as an effective intervention to improve sleep health among college students. **Methods:** Participants were undergraduate neuroscience majors (*n* = 11) enrolled in the Sleep and Circadian Rhythms capstone course at the University of St. Thomas. Over nine weeks, students engaged in a multidimensional analysis of their sleep behaviors. They completed a battery of assessments at the beginning and end of the semester, including the Epworth Sleepiness Scale (ESS), Pittsburgh Sleep Quality Index (PSQI), Insomnia Severity Index (ISI), Morningness-Eveningness Questionnaire (MEQ), and the Dysfunctional Beliefs and Attitudes about Sleep scale. In addition to completing these surveys, students tracked their sleep using wearable devices or smartphone applications. For one week, each student introduced an environmental or behavioral intervention aimed at improving—or intentionally disrupting—their sleep. Key metrics analyzed included average total sleep time, bedtime, wake time, sleep midpoint, schedule variability, and nap frequency. Morning and evening saliva samples were also collected to assess melatonin and cortisol levels, with a focus on dim light melatonin onset (DLMO) and cortisol awakening response (CAR). **Results:** Initial assessments showed an average ESS score of 10, a PSQI of 8.4, and an ISI of 6, indicating moderate sleep disturbances. MEQ results varied, with most students categorized as intermediate chronotypes. Social jetlag averaged 28.8 min, suggesting relatively minor shifts in sleep timing between weekdays and weekends. Weekday sleep patterns were generally more consistent, particularly in bedtime and sleep midpoint. Variability in sleep timing and duration ranged from 40 min to 1.5 h. Preliminary hormonal analyses, along with intra- and inter-individual variability in DLMO and CAR, will be presented. Pre- and post-course comparisons will also explore whether the course influenced students’ sleep behaviors, beliefs, and outcomes. **Conclusions:** Preliminary findings align with prior research showing college students often experience poor sleep quality and circadian misalignment, driven by academic pressures and inconsistent schedules. By integrating sleep science education with personal data tracking, this course may enhance students’ understanding of sleep health and encourage meaningful behavior change. Final reflections from students will shed light on the perceived impact of the course on their sleep awareness and habits.

## 24. Regularity of Sleep-Wake Timing and Risk of New-Onset Heart Rhythm Disorders: A Pre-Registered, Prospective Analysis of 69,725 UK Biobank Participants

**Authors**

Dr. Mark Czeisler-United States-Harvard Medical School

Dr. Josh Leota-Australia-Monash University

Ms. Flora Le-Australia-Monash University

Mr. Lachlan Cribb-Australia-Monash University

Mr. Beaudan Campbell-Brown-Australia-Monash University

Dr. Stephanie Yiallourou-Australia-Monash University

Mr. Andreas Kontopidis-United States-TH Chan School of Public Health

Dr. Shantha Rajaratnam-Australia-Monash University

Dr. Prashant Rao-United States-Beth Israel Deaconness Medical Center

Dr. Matthew Pase-Australia-Monash University

Dr. Daniel Kramer-United States-Beth Israel Deaconness Medical Center

**Abstract**

**Introduction:** Health implications of the consistency of sleep-wake timing remain exploratory, although emerging research has identified irregular sleep-wake timing as a potential risk factor for all-cause and cardiovascular mortality. Irregular sleep-wake timing co-occurs with and contributes to circadian misalignment, a potential risk factor for arrhythmogenesis, and recent laboratory studies in mice and humans have identified multiple mechanisms by which circadian rhythms regulate cardiac pacemaking activity. We investigated the relationship between accelerometer-measured sleep regularity and new-onset heart rhythm disorders, including cardiac arrhythmias and conduction disorders. **Methods:** In this pre-registered analysis, we analyzed data from a prospective UK Biobank cohort with 7-day accelerometer recordings between 2013–2015 and no diagnosed heart rhythm disorders up to one year after accelerometer recording. Timestamped sleep-wake estimates were used to calculate Sleep Regularity Index (SRI) scores, reflecting the average probability that participants were in the same state (awake or asleep) at timepoints separated by 24 h. Time-to-event analyses were performed for new-onset cardiac arrhythmias, conduction disorders, or either with SRI quintile as the predictor, time since accelerometer recording as the timescale, and for full adjustment: sex, age, ethnicity, employment, Townsend deprivation, sleep duration, physical activity, smoking pack-years, alcohol use, self-rated health, and pre-accelerometer comorbidities. **Results:** The primary analytic sample included 69,725 participants, of whom 39,980 (57.3%) were female and 30,981 (44.4%) were aged 60–69 years, followed for a median of 7.5 (IQR 6.9–8.0) years. Overall, 4318 (6.2%) participants experienced new-onset heart rhythm disorders. Compared to participants with highly regular sleep-wake schedules (80–100th percentile SRI), those in the highly irregular schedules (0–20th percentile SRI) had significantly higher risk of all outcomes (e.g., any heart rhythm disorder, hazard ratio 1.20 [95% CI 1.08–1.32] *p* = 0.0004). Post-hoc analysis reveals that the elevated risk was driven primarily by male participants with baseline cardiovascular disease. **Conclusions:** Minimizing highly irregular sleep-wake timing may reduce the risk of heart rhythm disorders, independent of sleep duration. Sleep regularity may be a practical modifiable behavioral risk factor to reduce arrhythmogenesis.

## 25. SUNSHINE—Light Exposure and Sleep-Wake Patterns in Parents of Infants Between Three and Nine Months Old

**Authors**

Ms. Vaida Verhoef-Netherlands-Human Technology Interaction, Industrial Engineering and Innovation Sciences, Eindhoven University of Technology, The Netherlands

Ms. Emma Visser-Netherlands-Human Technology Interaction, Industrial Engineering and Innovation Sciences, Eindhoven University of Technology, The Netherlands

Dr. Karin Smolders-Netherlands-Human Technology Interaction, Industrial Engineering and Innovation Sciences, Eindhoven University of Technology, The Netherlands

Dr. Niki Antypa-Netherlands-Department of Clinical Psychology, Faculty of Social Sciences, Leiden University, The Netherlands

Prof. Yvonne de Kort-Netherlands-Human Technology Interaction, Industrial Engineering and Innovation Sciences, Eindhoven University of Technology, The Netherlands

**Abstract**

**Background**—Sleep disturbances are common among parents of newborns due to the demands of infant care, often resulting in reduced and fragmented sleep and impaired daytime functioning in an emotionally intense phase. While it is generally understood that adequate light at the right time can improve sleep and stabilize circadian rhythms, the complex interactions between these factors in parents of young infants remain underexplored, especially on a day-to-day or within-day basis. **Methods**—The SUNSHINE study employs ambulatory measurements (actigraphy, light sensors, skin temperature buttons) and experience sampling methodology (ESM) to capture detailed data on sleep patterns, light exposure, mood, circadian rhythms, and daytime sleepiness in parents of children aged three to nine months over the course of two weeks. **Results**—We present the initial results of the SUNSHINE study, which includes an in-depth descriptive analysis of sleep–wake and light exposure patterns among parents of young infants. In this analysis, we examine the averages, ranges, and variances in light exposure both within and between participants, quantified using metrics such as average photopic lux, melanopic equivalent daylight illuminance (mEDI), time spent above specific light thresholds (10 lx, 100 lx, 250 lx, 1000 lx), and the mean timing of this exposure (MLiT). Sleep is assessed using both sleep diaries and actigraphy, and we report the average and variance in sleep timing, subjective sleep quality, efficiency, and fragmentation across the entire population as well as within individual participants. Additionally, we investigate the interdaily stability and intradaily variability of both light exposure and sleep–wake behavior. **Conclusions**—This paper presents a unique characterization of the circadian challenges experienced by parents of young infants and marks the first exploration into the interplay between light exposure and sleep patterns during this critical period.

**Keywords:** sleep-wake patterns; light exposure; circadian rhythms; parents

**Funding:** The study was funded by The LightCap project, which received support from the European Union’s Horizon 2020 research and innovation program under the Marie Sklodowska-Curie grant agreement No. 860613, and the BioClock project (number 1292.19.077) of the research program Dutch Research Agenda: Onderzoek op Routes door Consortia (NWA-ORC) which is (partly) financed by the Dutch Research Council (NWO).

## 26. Microglia-Specific Knockdown of the Circadian Clock Gene Bmal1 Increases Motility and Reduces Anxiety-like Behavior in Mice

**Authors**

Dr. Louise Ince-United States-University of Texas at Austin

Ms. Sophia Martinez-United States-University of Texas at Austin

Ms. Alekhya Vattikuti-United States-University of Texas at Austin

Ms. Anusha Dabak-United States-University of Texas at Austin

Ms. Emily Chan-United States-University of Texas at Austin

Ms. Lourdes Davis-United States-University of Texas at Austin

Dr. Andrew Gaudet-United States-University of Texas at Austin

Dr. Laura Fonken-United States-University of Texas at Austin

**Abstract**

Microglia are key cells regulating neuroimmune function, learning & memory, and behavior. Microglia activity varies throughout the day. On a molecular level, cellular circadian rhythms are generated by a transcription-translation feedback loop, which is synchronized by the central pacemaker in the brain. However, it remains unclear to what extent diurnal rhythms in microglial functions are driven by the cell-intrinsic clock within microglia themselves vs. cell-extrinsic cues. To analyze the effects of the cell-intrinsic circadian clock on microglia function, we generated mice with microglia-specific knockdown of the core clock gene Bmal1 (Bmal1flox/floxTmem119CreERT2, microglia-Bmal1KD). To assess the impact of Bmal1KD on microglia function, we isolated microglia from Bmal1KD and littermate controls to quantify inflammatory responses and phagocytic activity. Microglia isolated from Bmal1KD animals showed enhanced production of the inflammatory cytokine Il1b in response to immune stimulation via lipopolysaccharide (LPS), suggesting a change in the inflammatory activity of these cells. Furthermore, microglia isolated from Bmal1KD males, but not females, showed enhanced phagocytosis of fluorescent beads. Because microglia exhibited changes in functional activity, we next evaluated whether these changes altered behavioral outcomes. Microglia-Bmal1KD mice of both sexes showed elevated locomotor activity in the open field and elevated plus maze. Microglia-Bmal1KD also increase entries into the open arms and total time in the open arms compared to littermate controls. These results indicate that Bmal1 in microglia plays a previously unappreciated role in regulating motility and anxiety-like behavior in mice, and that these effects may be driven by altered neuroimmune signaling. Future work will focus on determining (1) how the anxiolytic and hyperkinetic effects of microglia-Bmal1KD are generated, (2) how sickness behavior is modulated by microglia-Bmal1KD, and (3) how Bmal1 modulates additional microglial functions such as synaptic pruning and diurnal rhythms in cell morphology.

## 27. Evening Blue Light Exposure During Adolescence Induces Avoidance Behaviors and Rewires Medial Amygdala Circuit

**Authors**

Dr. Pablo Bonilla Villamil-United States-University of South Carolina

Dr. Dave McBride-United States-University of California, San Diego

Ms. Alexandria Shanks-United States-University of South Carolina

Dr. Alessandra Porcu-United States-University of South Carolina

**Abstract**

**Background:** Currently more than 99% of the US and European populations experience light pollution. Recent studies have linked altered light environments to increased anxiety and mood disorders among U.S. adolescents. The medial amygdala (MeA), a key region regulating emotional response and anxiety behaviors, is one of several brain regions receiving direct projections from intrinsically photosensitive retinal ganglion cells (ipRGCs). These cells are more sensitive to blue light wavelengths, and blue light (compared to green) heightened responses to emotional stimuli and increases neuronal activity in the amygdala in humans. However, the impact of different wavelengths of light on the MeA circuitry and function during adolescence remain unknown. **Methods:** We recently established a translational light cycle disruption (LCD) paradigm to model the effects of irregular light exposure during adolescence. In this model, adolescent mice undergo a weekly schedule consisting of 12 h of light, 5 h of darkness, and 7 h of LED light at night for 5 consecutive days, followed by 2 days of standard 12L:12D conditions. This cycle is repeated for 4 weeks. One experimental group is exposed to broad-spectrum LED light during the 7-h night phase (LCD-BL), while another group receives light with reduced blue wavelengths during the same period (LCD-RBL). Control mice are maintained under a consistent 12L:12D cycle throughout the 4 weeks. Following light exposure, mice were assessed for anxiety-like behaviors, and brains were processed for single-nucleus RNA sequencing (snRNA-seq), immunohistochemistry, and RNA in situ hybridization (ISH) targeting the MeA. Fiber photometry was used to record real-time in vivo activity of somatostatin (SST) neurons in the MeA (MeA^SST^) during avoidance behaviors and chemogenetic manipulation was implemented to acutely inhibit MeA^SST^ neuronal activity following LCD exposure. **Results:** Behavioral analysis revealed that adolescent mice exposed to evening blue light (LCD-BL) showed increased avoidance compared to those exposed to reduced blue light (LCD-RBL) or control conditions. ScRNA-seq data showed differences in cell type composition, cell-cell communication and differential expression of genes involved in neuronal activity and synaptic function. We found that light-induced avoidance behavior was associated with increased cFOS expression (neuronal activity marker) selectively in somatostatin (SST) neurons in the MeA (MeA^SST^), but not in GABAergic or glutamate MeA neurons. In vivo calcium recordings demonstrated that mice exposed to LCD-BL exhibited increased MeA^SST^ neuronal activity associated with heightened avoidance behaviors compared to control. Selective chemogenetic inhibition of MeA^SST^ neurons following LCD-BL exposure was sufficient to rescue light-induced avoidance behaviors in adolescent mice. **Conclusions:** Our findings uncover key molecular, cellular, and behavioral changes associated with evening blue light exposure during adolescence. Furthermore, we establish the MeA as a critical hub integrating environmental light and emotional responses, advancing our understanding of how light exposure during sensitive developmental periods may impact emotional regulation. We suggest that chronic LCD with evening blue light, like those experienced by humans, might represent a risk factor for developing affective disorders.

**Funding:** NIH-NCCIH K99/R00AT010903 to A.P., R01AT013234 to A.P.

## 28. Wearable Light Dosimeter with RGB Sensors: A Practical Approach to Determine Melanopic EDI for Daylight Measures

**Authors**

Mr. Gunnar Deuring-Switzerland-Centre for Chronobiology, Psychiatric University Hospital, University of Basel, Basel, Switzerland.

Dr. Oliver Stefani-Switzerland-Lucern University of Applied Sciences and Arts.

Ms. Sophie Reckels-Switzerland-Centre for Chronobiology, Psychiatric University Hospital, University of Basel, Basel, Switzerland.

Ms. Johanna Otte-Switzerland-Centre for Chronobiology, Psychiatric University Hospital, University of Basel, Basel, Switzerland.

Dr. Christian Epple-Switzerland-Centre for Chronobiology, Psychiatric University Hospital, University of Basel, Basel, Switzerland.

Dr. Benedikt Reuthebuch-Switzerland-Centre for Chronobiology, Psychiatric University Hospital, University of Basel, Basel, Switzerland.

Dr. Corrado Garbazza-Switzerland-Centre for Chronobiology, Psychiatric University Hospital, University of Basel, Basel, Switzerland.

Dr. Martin Meyer-Switzerland-Psychiatric Hospital of the University of Basel, Basel, Switzerland.

Dr. Helen Slawik-Switzerland-Psychiatric Hospital of the University of Basel, Basel, Switzerland.

Prof. Christian Cajochen-Switzerland-Centre for Chronobiology, Psychiatric University Hospital, University of Basel, Basel, Switzerland.

Prof. Anne Eckert-Switzerland-Psychiatric Hospital of the University of Basel, Basel, Switzerland.

Prof. Aki Kawasaki-Switzerland-University of Lausanne, Switzerland.

Prof. Susanne Jaeggi-United States-Northeastern University, Boston, USA.

Dr. Mirjam Münch-Switzerland-Centre for Chronobiology, Psychiatric University Hospital, University of Basel, Basel, Switzerland.

**Abstract**

**(1) Background:** Circadian health interventions call for an assessment of circadian zeitgebers like physical activity, meal timing, and light exposure—the latter being the most powerful. The circadian synchronization effect of light is mainly mediated by the spectral range that excites the melanopsin-containing intrinsically photosensitive retinal ganglion cells (ipRGC). This pathway constitutes the non-image forming (melanopic) visual system. Among the various light sources we are exposed to in everyday life, it is natural daylight that exerts the most relevant effect on the melanopic system. When quantifying the effects of light on circadian rhythms, researchers usually refer to the melanopic equivalent daylight illuminance (mEDI). The mEDI of a lighting condition is defined as the illuminance of a standardized D65 daylight reference that would be required to produce the same activation of the melanopic pathway. Wearable light dosimeters can be employed to attain an economical measure of mEDI. These devices are usually equipped with sensors for photopic illuminance and preferably also for at least the red, green, and blue spectral portions (RGB) of a given light source. However, calculating an estimate for mEDI directly from the photopic lux readings is only more or less appropriate for close to D65 daylight situations, which is rarely the case in real life. **(2) Methods:** We propose a differentiated approach to estimate mEDI utilizing the dosimeter’s RGB sensor information to infer on current spectral properties and further derive a correction factor, i.e., the melanopic daylight efficacy ratio (MDER), to attain an accurate estimate of mEDI from photopic illuminance across a range of daylight conditions. Instead of operating on the premises of a perceptional color space model we are applying principles from the Compositional Data Analysis framework and non-linear regression techniques. The relative RGB proportions of measured samples are transformed to an isometric log-ratio space (ILR) and MDER is calculated from the ILR location with reference to two gradient lines, which are defined by the known MDER values of the Planckian locus and the CIE D-series standard daylight illuminants (D50-D75). **(3) Results:** Although the information of the RGB sensors in theory cannot always unambiguously separate different spectra, this approach showed that within the broad range of daylight conditions the resulting mEDI estimates are more differentiated and represent the real conditions more accurately than just using photopic illuminance as surrogate. **(4) Conclusions:** This procedure was developed with data from the wearable ActTrust 2 light sensor devices (Condor Instruments, São Paulo, Brazil) and can be transferred to any similar RGB sensor devices where RGB readings are available in a standardized unit and the RGB sensitivity curves provided. The possibility of estimating mEDI from inexpensive light dosimeters will greatly facilitate research on the circadian light effects and applied interventions.

**Kewords:** Circadian health; mEDI; light sensors

**Funding:** Velux Stiftung, Switzerland.

## 29. Food Timing, Siesta, and Obesity: How Meal Schedules and Post-Meal Naps Influence Body Weight

**Author**

Prof. Marta Garaulet-Spain-Department of Physiology, University of Murcia

**Abstract**

In recent years, the global rise in obesity has drawn increasing attention to the complex interplay between lifestyle factors and metabolic health. While much of the discourse has centered on calorie intake and physical activity, emerging evidence suggests that the timing of food consumption and sleep patterns—particularly siesta behavior—may also play a critical role in energy balance and body weight regulation. In this lecture, I will explain the interrelation between food timing, postprandial (after-meal) naps, and obesity, aiming to provide a nuanced understanding of how circadian rhythms and cultural practices intersect to influence metabolic outcomes. Meal timing, often overlooked in traditional nutritional assessments, is increasingly recognized as a significant modulator of metabolic processes. The when we eat may be as crucial as what we eat. Eating during the biological night, for instance, is associated with impaired glucose tolerance, decreased insulin sensitivity, and greater fat storage. Conversely, early time-restricted feeding (e.g., consuming meals within an 8–10-h window during daylight) has been shown to improve weight management and cardiometabolic health markers. The role of the siesta—a common cultural practice in many Mediterranean and Latin American countries—characterized by a short nap taken shortly after lunch. While historically perceived as restorative, the metabolic consequences of siesta habits remain understudied and somewhat controversial. Our investigation examines whether siestas after large midday meals contribute to increased adiposity. Our results underscore the importance of synchronizing eating and resting behaviors with circadian biology. public health strategies aiming to combat obesity may benefit from incorporating chronobiological insights into dietary guidelines. Second, culturally sensitive interventions are needed to educate communities about optimal meal and rest timing without undermining valuable social traditions such as the siesta. In conclusion, our findings advocate for a more holistic approach to obesity prevention—one that respects cultural practices while promoting metabolic health through strategic meal timing and conscious rest patterns. Future longitudinal studies and randomized trials are warranted to further disentangle causality and refine recommendations.

## 30. Biological and Clinical Insights from Genetics of Circadian Rhythms

**Author**

Dr. Jackie Lane-United States-Division of Sleep and Circadian Disorders, Departments of Medicine and Neurology, Brigham and Women’s Hospital

**Abstract**

Being a morning person is a behavioral indicator of a person’s underlying circadian rhythm. Using genome-wide data from 697,828 UK Biobank and 23andMe participants we increase the number of genetic loci associated with being a morning person from 24 to 351. The loci are enriched for genes involved in circadian regulation, cAMP, glutamate and insulin signaling pathways, and those expressed in the retina, hindbrain, hypothalamus, and pituitary. Using Mendelian Randomization, we show that being a morning person is causally associated with better mental health. Next we investigated whether chronotype per se it causally for mental health, or rather is mediated by circadian mistiming often seen in evening types. Using a measure of sleep inertia we find that evening chronotypes who awaken during the biological night have a higher risk of psychiatric disorders compared to those who awaken at an appropriate time. Furthermore, we have followed up on several of the chronotype loci to determine the likely functional variants in the regions.

## 31. Self-Directed, Fully-Remote Circadian Phenotyping to Address the Needs of a Diverse Patient Population with Circadian Rhythm Disorders

**Author**

Dr. Jackie Lane-United States-Division of Sleep and Circadian Disorders, Departments of Medicine and Neurology, Brigham and Women’s Hospital

**Abstract**

**Study Objectives** To test the feasibility of a novel at-home self-directed salivary Dim Light Melatonin Onset (DLMO) assessment protocol to measure endogenous circadian phase. **Methods** The 5–6 week protocol involved sleep, activity, and light tracking, self-reported sleep diaries, the morningness-eveningness questionnaire, and two self-directed home DLMO collections one week apart with objective compliance measures. Individuals participated remotely using an online portal and a mailed a kit with all materials for actigraphy and at-home sample collections. The study population was 5 cases with circadian rhythm disorders and 5 controls ages 27–63 (*n* = 1 advanced sleep wake phase disorder ASWPD, *n* = 4 delayed sleep wake phase disorder DSWPD, *n* = 5 controls). 7 individuals self-identified as Caucasian and 3 self-identified as Asian. Diverse gender identities were reported (woman = 6, male = 1, transgender = 1, nonbinary = 1, none = 1). **Results** Salivary DLMO times could be calculated for 8/10 participants. Of the remaining 2 participants, one was non-compliant with lighting levels during the DLMO collection and the other demonstrated consistently low levels of melatonin secretion during the collection window and was referred for a 24-h urinary melatonin collection. DLMO times were on average 2.63 h earlier than self-reported bedtime in DSWPD participants, and 3.413 h earlier than self-reported bedtime in controls. DLMOs 1 and 2 were strongly correlated (r = 0.96, *p* < 0.005). **Conclusions** Our results indicate that self-directed, at-home DLMO assessments are feasible and precise. The current protocol may serve as a framework to reliably assess circadian phase in both clinical and general populations.

## 32. Circadian Regulation of Neuroinflammation: Implications for Brain and Behavior

**Author**

Dr. Laura Fonken-United States-University of Texas at Austin

**Abstract**

Activation of the immune system changes mammalian behavior. Pathogens and injury cause peripheral immune cells to send signals to the brain, which act on immune cells in the brain such as microglia. Upon receiving peripheral immune signals, microglia begin secreting inflammatory molecules in the central nervous system, ultimately inducing sickness behaviors (e.g., lethargy, social withdrawal, and cognitive impairments). Importantly, pathways that confer this adaptive sickness response are non-specific, so aberrant microglia activity outside the context of sickness can also alter behavior. This talk will explore circadian regulation of microglia function, and how the link between the circadian system and microglia can impact behavioral responses. Indeed, our work has demonstrated that microglia exhibit time-of-day differences in their functions, such as changes in inflammatory cytokine production and phagocytic activity. Disruption of the circadian system, by environmental or genetic perturbation, can alter microglia activity leading to changes in behavior. Moreover, rhythms in microglia activity dampen with age and are associated with elevated neuroinflammation. Bolstering circadian rhythms with time-restricted feeding can abrogate these age-associated neuroinflammatory and behavioral changes. Overall, our data suggest that targeted circadian-based strategies could benefit neuroimmune and behavioral pathologies.

## 33. Ground and Flight Studies Testing the Visual and Physiological Effects of Tunable LED Lighting for the International Space Station (ISS)

**Authors**

Dr. George Brainard-United States-Light Research Program, Department of Neurology, Thomas Jefferson University

Dr. John Hanifin-United States-Light Research Program, Department of Neurology, Thomas Jefferson University

Dr. Mijail Serruya-United States-Thomas Jefferson University

Mr. Benjamin Warfield-United States-Light Research Program, Department of Neurology, Thomas Jefferson University

Mr. John Kemp-United States-Light Research Program, Department of Neurology, Thomas Jefferson University

Mr. Taehwan Yoo-United States-Light Research Program, Department of Neurology, Thomas Jefferson University

Mr. Robert Soler-United States-BIOS Lighting

Dr. Shadab Rahman-United States-Harvard Medical School

Dr. Melissa St. Hilaire-United States-Department of Computer and Data Sciences, Merrimack College

Ms. Toni Clark-United States-NASA Habitability and Human Factors Branch

Ms. Kelly Norwood-United States-NASA Research Operations and Integration

Mr. Daniel Garcia-United States-NASA Research Operations and Integration

Dr. Smith Johnston-United States-NASA Fatigue Management Team for Space Flight Operations

Dr. Ronald Moomaw-United States-NASA Outpatient Behavioral Health

Dr. Charles Czeisler-United States-Division of Sleep and Circadian Disorders, Departments of Medicine and Neurology, Brigham and Women’s Hospital; Division of Sleep Medicine, Harvard Medical School

Dr. Laura Barger-United States-Division of Sleep and Circadian Disorders, Departments of Medicine and Neurology, Brigham and Women’s Hospital; Division of Sleep Medicine, Harvard Medical School

Dr. Steven Lockley-United States-Division of Sleep and Circadian Disorders, Departments of Medicine and Neurology, Brigham and Women’s Hospital; Division of Sleep Medicine, Harvard Medical School

**Abstract**

**Background:** On space shuttle and ISS missions, astronauts slept about one hour less on nights when circadian misaligned [Flynn-Evans et al. (2016) *npj Micrograv*] [1]. Light can be a powerful countermeasure for both circadian misalignment and sleepiness. The ISS was originally equipped with fluorescent lighting for illuminating the astronauts’ working and living environments. Those lights have been replaced with Solid State Lighting Assemblies (SSLAs) [NASA Specification (2013) S684-13489]. The SSLAs have three light settings, each with a unique intensity and spectrum: (1) General Illumination; (2) Alertness/Phase Shift; and (3) Pre-Sleep. The aim of this work was to test light emitted by SSLAs for supporting astronaut visual performance, color discernment, circadian, neuroendocrine, and sleep physiology as well as neurobehavioral functioning. **Methods:** A dynamic lighting schedule was developed based on the spectral and intensity sensitivities of the human circadian photoreceptor system. The first aim was to conduct three ground analog studies using healthy astronaut-aged volunteers: a 5-day, controlled inpatient study testing the efficacy of a lighting protocol for daily operations utilizing SSLAs and two studies on color vision and visual performance. Those studies were conducted in the high-fidelity ISS analog crew sleeping quarters. The second aim was to conduct an in-flight study on the ISS testing the efficacy of the lighting protocol for daily operations using SSLAs. Data from these studies led to a follow-up analog study testing a modification of the SSLA Pre-Sleep setting. Nine healthy astronaut-aged men and women participated in a randomized, three condition study involving SSLA light exposures of General Vision, the original Pre-Sleep setting, and a new Pre-Sleep setting modified in irradiance and wavelength. These light exposures took place in the replica of the ISS crew sleeping quarters. Dependent variables included dim light melatonin onset (DLMO) and the Farnsworth Munsell 100 (FM100) color vision test. **Results:** Ground analog studies showed significantly earlier onset of melatonin under a dynamic light schedule compared to a static light schedule (*p* < 0.005). Analog studies and the ISS flight study showed color vision was significantly decreased under the dim Pre-Sleep setting (*p* < 0.001). The current ground analog study has shown that exposure to both Pre-Sleep settings resulted in significantly greater production of melatonin compared to the General Vision setting (*n* = 8, df = 2, *p* < 0.03). Compared to General Vision, color vision was significantly compromised under both Pre-Sleep settings (*p* < 0.05). Data analysis is ongoing in both analog and flight studies. **Conclusions:** Risk factors for the health and safety of astronauts include disturbed circadian rhythms and altered sleep-wake patterns. Ultimately, study results will determine if SSLA lighting can support astronaut vision and serve as an in-flight countermeasure for circadian misalignment, sleep disruption and performance deficits on ISS flight missions.

**Keywords:** light; melatonin; dynamic lighting; color vision; spaceflight

**Support:** Primary funding: NASA #NNX15AC14G. Additional support: NSBRI through NASA NCC 9-58, and the Nova Institute for Health.

**References**

1. Flynn-Evans, E.E.; Barger, L.K.; Kubey, A.A.; Sullivan, J.P.; Czeisler, C.A. Circadian misalignment affects sleep and medication use before and during spaceflight. *npj Microgravity* **2016**, *2*, 1–6.

## 34. Effects of an Integrated Sleep and Circadian Intervention on Reward Sensitivity and Impulsivity Metrics in Adolescents with Delayed Sleep Timing

**Authors**

Dr. Delainey Wescott-United States-University of Pittsburgh

Dr. Daniel Buysse-United States-University of Pittsburgh

Dr. Duncan Clark-United States-University of Pittsburgh

Dr. Greg Siegle-United States-University of Pittsburgh

Dr. Meredith Wallace-United States-University of Pittsburgh

Ms. Nina Oryshkewych-United States-University of Pittsburgh

Ms. Allysa Quick-United States-University of Pittsburgh

Ms. Riya Mirchandaney-United States-University of Pittsburgh

Dr. Brant Hasler-United States-University of Pittsburgh

**Abstract**

Delayed sleep timing coupled with early school start times lead to insufficient and misaligned sleep during adolescence. Short sleep and circadian misalignment can negatively impact reward sensitivity and impulsivity, processes implicated in substance use behaviors. We tested whether an integrated sleep/circadian intervention advanced and extended sleep and modified reward sensitivity and impulsivity. We also determined if advanced sleep/circadian timing were associated with changes in reward sensitivity and/or impulsivity. Following an 8-day baseline period, 80 high school juniors and seniors (ages 16–19, 60% female sex at birth) were randomized to a 2-week sleep/circadian manipulation (*n* = 40) or a sleep monitoring control (*n* = 40). The manipulation included stable wake times, increased morning light (Re-Timers), decreased evening light (blue-blocking glasses), and advanced weeknight bedtimes. Circadian phase was measured using salivary dim light melatonin onset (4 pg/mL). Sleep was measured continuously by actigraphy. Reward and impulsivity metrics were captured using self-report (Behavioral Activation Scale [BAS]; UPPS-P Impulsivity Scale) and behavioral tasks (Balloon Analogue Risk Task [BART], Cued Go/No-Go Task [CGNG]). Substance use was assessed using a Timeline Followback Interview. Multilevel models compared sleep, circadian, and reward/impulsivity related changes between conditions across time, and tested associations between sleep/circadian and reward/impulsivity changes controlling for sex at birth. At baseline, 18.8% of participants reported cannabis use and 36.3% reported alcohol use during the past 3-months. The sleep/circadian manipulation advanced circadian phase by ~36-min (B = −0.55; *p* < 0.001), extended weeknight sleep by ~40-min (B = 0.76; *p* = 0.001), and prevented an increase in behavioral reward motivation (BART: B = −0.43; *p* = 0.018) relative to the control condition. Advances in circadian phase were associated with reductions in behavioral reward motivation (BART: B = 0.21; *p* = 0.028). Extended weeknight sleep was associated with decreased lack of perseverance (UPPS-P: B = −0.21; *p* = 0.003) and increased reward responsiveness (BAS: B = 0.20; *p* = 0.046). A 2-week integrated sleep/circadian intervention advanced circadian phase and extended weeknight sleep for adolescents with delayed sleep timing who may be at risk for engaging in substance use. Despite relatively low rates of substance use in the current sample, our findings support the hypothesis that modifying sleep/circadian factors can, in turn, impact reward sensitivity and impulsivity. Longer-term interventions may be necessary to test if sleep and circadian changes can be maintained and whether changes in reward sensitivity and impulsivity reduce future substance use.

## 35. The Microglial Clock Contributes to Sex-Specific Changes in Neurodevelopment

**Authors**

Mrs. Brandy Routh-United States-University of Texas at Austin

Ms. Cecily Gibson-United States-University of Texas at Austin

Ms. Celina Yang-United States-University of Texas at Austin

Ms. Leanna Doan-United States-University of Texas at Austin

Dr. Andrew Gaudet-United States-University of Texas at Austin

Dr. Hans Hofmann-United States-University of Texas at Austin

Dr. Regina Mangieri-United States-University of Texas at Austin

Dr. Laura Fonken-United States-University of Texas at Austin

**Abstract**

Children in modern society face an unprecedented level of circadian rhythm disruption from nighttime smartphone use and urban light pollution, among other factors. While circadian disruptions are associated with poor mental health, the mechanisms by which the circadian system may intersect with or influence neurodevelopmental processes remain unclear. Adolescence comprises a critical developmental window during which synaptic connectivity is scaled back in a process called developmental synaptic pruning, and microglia, the brain’s resident macrophages, participate in this process by phagocytosing synaptic elements. Adult microglia exhibit diurnal rhythms in phagocytosis, so here, we hypothesized that early-life disruption of microglia rhythms could alter developmental synaptic pruning and impair the anatomical and physiological refinement of developing neural circuits. To test this, we disrupted the microglia molecular clock in a mouse model with tamoxifen-inducible, microglia-specific, deletion of the essential circadian gene, Bmal1. After tamoxifen injection on postnatal days (pnd) 3–5, we compared Bmal1 cKO^MG^ mice to wildtype littermate controls in early adolescence (pnd 28–40) using a combination of transcriptomics, immunohistochemistry, and slice electrophysiology. In wildtype microglia, synaptic pruning gene expression peaked during the animals’ rest phase, but this time-of-day dependent expression was abolished in Bmal1 cKO^MG^ mice. Male, but not female, Bmal1 cKO^MG^ microglia displayed enhanced synaptic engulfment and reduced density of dendritic spines, which represent the anatomical correlate of excitatory synapses. Both male and female Bmal1 cKO^MG^ mice had reduced amplitudes of evoked synaptic currents in hippocampal CA1 pyramidal neurons, indicating a functional reduction in synaptic connectivity in both sexes. Bulk hippocampal RNA sequencing revealed differential gene expression in pathways regulating pre- and postsynaptic organization in Bmal1 cKO^MG^ mice, with additional impacts to postsynaptic plasticity pathways specifically in male Bmal1 cKO^MG^ mice. These data uncover the microglial clock as a critical regulator of developmental synaptic refinement, providing a novel mechanism connecting early-life circadian rhythms with the sculpting of the adolescent brain, with broad implications for pediatric health in the modern world.

**Funding sources:** NSF GRFP to BR and NIH R01AG078758 to LKF.

## 36. Temporal Dynamics of Circadian Rhythms and Sleep-Wake Patterns in Breast Cancer: From Diagnosis to 12-Month Follow-Up

**Authors**

Dr. Ali Amidi-Denmark-Sleep & Circadian Psychology Research Unit, Department of Psychology and Behavioural Sciences, Aarhus University, Aarhus, Denmark

Dr. Ru Li-Denmark-Sleep & Circadian Psychology Research Unit, Department of Psychology and Behavioural Sciences, Aarhus University, Aarhus, Denmark

Dr. Dinne Christensen-Denmark-Department of Psychology & Behavioral Sciences, Aarhus University

Prof. Robert Zachariae-Denmark-Unit for Psychooncology & Health Psychology, Aarhus University Hospital

Prof. Peer Christiansen-Denmark-Department of Plastic and Breast Surgery, Aarhus University Hospital

Prof. Anders Bonde-Denmark-Department of Clinical Medicine, Aarhus University

Prof. Frank Penedo-United States-Departments of Psychology and Medicine, University of Miami Miller School of Medicine

Prof. Sonia Ancoli-Israel-United States-Department of Psychiatry, University of California San Diego School of Medicine

Prof. Kathryn Reid-United States-Center for Circadian and Sleep Medicine, Northwestern University, Chicago, USA

Dr. Lisa Wu-Denmark-Sleep & Circadian Psychology Research Unit, Department of Psychology and Behavioural Sciences, Aarhus University, and Department of Psychology, Reykjavik University, Reykjavik, Iceland

**Abstract**

**Background**: Breast cancer (BC) and its treatment are associated with behavioral and psychological late effects, including sleep disturbances with an estimated prevalence of 50%. Although the exact causes and underlying pathophysiology of disturbed sleep remain unclear, we have recently hypothesized that treatment-related circadian disruption may play a role. Therefore, the aim of the present study was to longitudinally investigate changes in markers of circadian rhythms and their association with actigraphy-based sleep in BC patients compared with matched healthy controls (HCs). Trial registration: NCT04401189. **Methods**: Patients newly diagnosed with BC were enrolled before any cancer treatments. Participants were scheduled for a total of four assessment time points: Baseline (T1, before treatment), after surgery or neoadjuvant chemotherapy (T2), after completion of adjuvant chemotherapy and/or surgery (T3), and at 12-month follow-up (T4). Age-matched healthy controls (HCs) were assessed at comparable intervals. Data from T1, T3 (post-primary treatment), and T4 were included in the analyses. Circadian activity rhythms and actigraphy-based sleep were assessed using the ActTrust actigraph (Condor Instruments, São Paulo, Brazil), which was worn for seven consecutive days at each time point. Calculated variables included the Circadian Function Index (CFI), L5 time, bedtime, get-up time, total sleep time (TST), sleep efficiency (SE), sleep onset latency (SOL), wake after sleep onset (WASO), and number of nighttime awakenings (NA). Dim-light melatonin onset (DLMO) was assessed in patients at T1 and T4 only. Seven hourly saliva samples were collected starting six hours before habitual bedtime while wearing blue light blocking glasses (LowBlueLights™ Traditionalists). DLMO was determined as the mean + 2 SD of the first three assessment points. Linear mixed-effects models (LMMs) were used to evaluate changes in outcomes over time and group × time interactions. **Results**: Statistically significant group differences over time were observed between BC patients (*n* = 56) and HCs (*n* = 65) in L5 phase (*p* = 0.026), get-up time (*p* < 0.001), TST (*p* = 0.002), WASO (*p* = 0.013), and NA (*p* = 0.002), indicating progressive circadian delay and worsening sleep continuity in the BC group. A marginal difference in SE at T3 was noted (*p* = 0.079). No significant interaction effects were found for SOL, bedtime, or CFI. Among patients (*n* = 28), DLMO advanced significantly by 0.78 h from T1 (*p* = 0.003). A greater DLMO advance was associated with later get-up time (β = 0.24, *p* < 0.001), longer TST (β = 0.26, *p* < 0.001), and better SE (β = 0.10, *p* = 0.030), as well as longer SOL (β = 0.15, *p* = 0.005), and greater WASO (β = 0.11, *p* = 0.006), and NA (β = 0.11, *p* = 0.009). A marginal association was also found for CFI (β = −0.116, *p* = 0.083). At T1, mean CFI was marginally associated with lower WASO (β = −5.39, *p* = 0.058) and higher SE (β = 4.83, *p* = 0.054), indicating better sleep continuity among individuals with more robust circadian rhythms. However, CFI did not significantly predict change trajectories over time. Time-specific interaction analysis showed that a higher CFI was associated with a phase delay in L5 at T3 (β = 0.37, *p* = 0.014). **Conclusions**: These results suggest that BC and its treatments are associated with progressive changes in circadian markers and worsening sleep continuity. Notably, patients exhibited a significant advancement in DLMO, which was linked to both deteriorations (increased sleep fragmentation) and improvements (higher TST and SE) in sleep parameters. These improvements appeared to be mainly driven by later get-up times. Taken together, these findings suggest a complex interplay between circadian rhythms and sleep disturbances, which may be a future target for interventions.

**Keywords:** sleep; circadian rhythms; breast cancer; actigraphy; melatonin

**Funding:** Danish Cancer Society #R174-A11447-17-S52.

## 37. Dynamics and Ultradian Structure of Sleep in Patients with Seasonal Depression

**Authors**

Dr. Melissa St Hilaire-United States-Department of Computer and Data Sciences, School of Engineering and Computational Sciences, Merrimack College, North Andover, MA and Division of Sleep Medicine, Harvard Medical School, Boston, MA

Dr. Janis Anderson-United States-Corresponding Member of the Faculty of Psychology, Department of Psychiatry, Harvard Medical School; Scholar, Brigham & Women’s Hospital, Boston, MA and Division of Sleep Medicine, Harvard Medical School, Boston, MA

**Abstract**

**Background:** Sleep disturbances are a prominent feature of Major Depression, Recurrent Seasonal Pattern (SAD) during depressive episodes [1]. However, most individuals with SAD are outpatients, and relatively little polysomnography (PSG) data are available. Many studies, however, have employed actigraphy. Here, we evaluate the utility of the Locomotor Inactivity During Sleep (LIDS) method to characterize the dynamics and ultradian structure of sleep from actigraphy data among individuals during an episode of SAD prior to receiving light treatment. **Methods:** Fifty-six medication-free subjects aged 21–64 years who met DSM-IV-TR criteria for recurrent major depression with winter-type seasonal pattern were originally enrolled in a blinded study at five participating centers between January and March 2012 [2]. Twenty-nine subjects completed the study. Actigraphy (Actiwatch Spectrum, Philips Healthcare) was collected for one baseline week and through 6 weeks of treatment, and sleep bouts were identified using the Phillips Actiware software (6.3 version). LIDS was calculated by transforming 10-min binned activity counts during sleep using the LIDS formula provided by Winnebeck et al. [3]. The LIDS signal was smoothed using a centered 30-min rolling average and Z-scored within each sleep bout. Ultradian rhythm metrics were extracted using Fast Fourier Transform (FFT) to identify the dominant frequency within a 30–240 min band, and a cosine model was fit at that period to estimate the amplitude and phase of the ultradian oscillation. The analysis included only nighttime sleep bouts that were longer than 3 h. Only participants for whom we had at least 5 nights of actigraphy during the baseline week of the study were included in the present analysis. **Results:** Across all sleep bouts collected during the baseline week (*n* = 178; median, IQR = 7.84 h, 6.76–8.78 h) in *n* = 25 individuals with SAD, the median period of the LIDS rhythm was 106.75 min (IQR: 85.79–154.58 min). The median phase of the ultradian rhythm, which represents the phase after sleep onset when the first peak in inactivity occurs, was 188 degrees (IQR: 146–229 degrees), indicating that the peak inactivity occurred around the midpoint of the ultradian cycle. The median amplitude was 0.69 (IQR: 0.57–0.79), indicating strong ultradian rhythms. The median relative ultradian power was 0.69 (IQR: 0.55–0.79), another indicator of strong ultradian rhythms. **Conclusions:** The median period of the LIDS rhythm in our SAD cohort was in the same range of 105–110 min observed in a general population analysis^3^. The median LIDS phase at sleep onset was 188 degrees, indicating that the first peak in inactivity occurred approximately halfway through the ultradian cycle (i.e., ~55 min into a 106.25-min cycle), which is consistent with PSG-reported observations of deep NREM sleep peaking 45–60 min after sleep onset. Together these results suggest no differences in the ultradian sleep-wake cycles in our cohort of individuals with SAD compared with the general population. Future LIDS analysis should focus on assessing ultradian sleep-wake rhythms closer to the onset of SAD symptoms, i.e., in autumn, as well as examining the longitudinal effect of SAD treatment on ultradian cycles.

**Funding:** The original treatment trial was funded by Philips HealthCare Solutions and NIH 1 UL1 RR025758-04 to the Harvard Clinical and Translational Science Center from the National Center for Research Resources. MSH was supported by T32 HL079010.

**References**

1. Anderson, J.L.; Rosen, L.N.; Mendelson, W.B.; Jacobsen, F.M.; Skwerer, R.G.; Joseph-Vanderpool, J.R.; Duncan, C.C.; Wehr, T.A.; Rosenthal, N.E. Sleep in fall/winter seasonal affective disorder: Effects of light and changing seasons. *J. Psychosom. Res*. **1994**, *38*, 323–337.
2. Anderson, J.L.; St Hilaire, M.A.; Auger, R.R.; Glod, C.A.; Crow, S.J.; Rivera, A.N.; Fuentes Salgado, S.M.; Pullen, S.J.; Kaufman, T.K.; Selby, A.J.; et al. Are short (blue) wavelengths necessary for light treatment of seasonal affective disorder? *Chronobiol. Int*. **2016**, *33*, 1267–1279.
3. Winnebeck, E.C.; Fischer, D.; Leise, T.; Roenneberg, T. Dynamics and Ultradian Structure of Human Sleep in Real Life. *Curr. Biol*. **2018**, *28*, 49–59.e5.

## 38. Use of Apple Watch to Optimize Light Therapy and Reduce Circadian Misalignment for Night Shift Workers

**Authors**

Dr. Philip Cheng-United States-Henry Ford Health + Michigan State University Health Sciences

Dr. Olivia Walch-United States-Arcascope

Mr. Marleigh Treger-United States-Henry Ford Health + Michigan State University Health Sciences

Dr. Christopher Drake-United States-Henry Ford Health + Michigan State University Health Sciences

**Abstract**

**Introduction:** Circadian misalignment can be alleviated with targeted light interventions. However, existing approaches do not account for shift workers’ variable circadian phases. Recently, we have validated the use of wearable data from the Apple Watch (AW) to predicting circadian timing (i.e., dim light melatonin onset, DLMO). Here, we extend this by examining the clinical utility of AW in informing circadian interventions. We hypothesized the AW-informed group would result in higher rates of circadian alignment following treatment vs. the control (non-personalized) group. We also compared the magnitude of phase shifts between these interventions. **Methods:** Participants (*n* = 46) were randomly assigned to either the AW or control group, and DLMO was measured before and after treatment. AW data (accelerometer and heart rate) was collected over 2 weeks and processed through a biomathematical model of the human circadian system to produce estimated DLMOs. Light therapy schedules were created from the corresponding phase response curves and implemented with light boxes and blue-blocker glasses (at home or in lab) to induce phase shifts with the goal of circadian alignment. Participants in the control group followed a non-personalized light schedule (light from 18:00 and 21:00 and light avoidance from 04:00 and 10:00). Circadian alignment was operationalized as DLMO between 02:00 and 14:00, and a relative risk ratio was used to compare the rate of circadian alignment between the two groups. **Results:** The rate of circadian alignment post-treatment was 2.6 times higher in the AW group (56.8%) compared to the control group (22.2%). Additionally, those in the AW group achieved phase delays that were 8.5-times greater than the control group (AW group: delay of 2.5 h ± 5.0 SD; control group: 0.3 h ± 4.0 SD). **Conclusions:** These findings support the use of AW as a non-invasive method of generating personalized light treatments. Accessible and effective circadian treatments are key to improving the safety of nightshift workers. In the future we aim to perform sensitivity analyses and compare the efficacy of personalized light interventions at home versus in lab, to establish the feasibility of prescribing personalized light therapy as an at-home treatment option.

## 39. Effect of Three Weeks of Chronic Circadian Disruption Combined with Sleep Restriction on Heart Rate Variability in Healthy Young Adults

**Authors**

Dr. Robin Yuan-United States-Division of Sleep and Circadian Disorders, Departments of Medicine and Neurology, Brigham and Women’s Hospital; Division of Sleep Medicine, Harvard Medical School

Dr. Kirsi-Marja Zitting-United States-Division of Sleep and Circadian Disorders, Departments of Medicine and Neurology, Brigham and Women’s Hospital; Division of Sleep Medicine, Harvard Medical School

Dr. Jeanne Duffy-United States-Division of Sleep and Circadian Disorders, Departments of Medicine and Neurology, Brigham and Women’s Hospital; Division of Sleep Medicine, Harvard Medical School

Dr. charles czeisler-United States-Division of Sleep and Circadian Disorders, Departments of Medicine and Neurology, Brigham and Women’s Hospital; Division of Sleep Medicine, Harvard Medical School

Dr. Kun Hu-United States-Department of Anesthesia, Critical Care, and Pain Medicine, Massachusetts General Hospital; Division of Sleep Medicine, Harvard Medical School

**Abstract**

**Background:** Heart rate variability (HRV) provides a non-invasive assessment of autonomic cardiac control. Previous studies have identified an endogenous circadian rhythm in HRV. We aimed to test whether the circadian HRV rhythm changed after exposure to chronic circadian disruption combined with sleep restriction. **Methods:** Twelve healthy adults (18–27 yrs) participated in a 3-week inpatient study wherein 28-hr sleep-wake cycles with 6.5 h for each sleep period (5.6 h/24 h) were scheduled. Electrocardiography (ECG) was recorded continuously and used to derive R-R intervals (time interval between two consecutive R waves). HRV analysis was performed on 10-min segments according to the standards of the Task Force to obtain mean R-R interval, root mean square of successive differences (RMSSD), and normalized high-frequency (0.15–0.40 Hz) power (HFnu) —a measure of parasympathetic activity. A circadian phase (60° bins) determined by core body temperature was assigned to each segment. Mixed model analyses were performed separately for scheduled wake and sleep episodes, with HRV measures as outcomes, circadian phase, exposure week, and their interaction as fixed factors, and subject as a random factor. **Results:** During sleep, we observed a main effect of circadian phase on mean R-R interval, RMSSD, and HFnu (*p* < 0.0001 for all). In week 1, the circadian HRV rhythms peaked at ~300° and reached a nadir at ~120° for mean R-R interval, RMSSD, and HFnu. We also observed a main effect of exposure week on mean R-R interval and HFnu (*p* < 0.0001 for both), and an interaction between exposure week and phase for mean R-R interval, RMSSD, and HFnu (*p* < 0.001 for all). Compared to week 1, mean R-R interval was 3–4% higher during week 3 at ~60° and ~120° (adj. *p* < 0.0001); RMSSD was 7–10% higher during week 3 at ~60° and ~120° (adj. *p* < 0.001); and HFnu was 6–11% higher during week 3 at ~60°, 120°, and 180° (adj. *p* < 0.01), resulting in a dampening of the overall rhythms. The rhythms of both HFnu and RMSSD also appeared to phase advance by ~120° between week 1 and 3, i.e., they peaked at ~180° and reached a nadir at ~0° in week 3. During wakefulness, we observed a main effect of circadian phase on mean R-R interval, RMSSD, and HFnu (*p* < 0.0001 for all). In week 1, the rhythms peaked at ~0° and reached a nadir at ~240° for mean R-R interval, RMSSD, and HFnu. We also observed a main effect of exposure week on mean R-R interval (*p* = 0.02) and RMSSD (*p* < 0.0001), as well as an interaction between exposure week and phase for mean R-R interval (*p* < 0.0001), RMSSD (*p* = 0.01), and HFnu (*p* = 0.004). HFnu was 4–5% lower at ~0° and 120° during week 3 compared to week 1 (adj. *p* < 0.05 for both) and appeared to phase advance ~120° between week 1 and week 3. RMSSD was 5–7% lower at all circadian phases during wakefulness in week 3 compared to week 1 (adj. *p* < 0.001 for all). **Conclusions:** Chronic exposure to circadian disruption combined with sleep restriction led to dampened circadian HRV rhythms with increased parasympathetic activity and decreased heart rate during the circadian phases corresponding to late morning afternoon hours, which might reflect a compensatory change in autonomic control.

**Funding:** This work was supported by the American Academy of Sleep Medicine (Focused Projects Grant for Junior Investigators) and grants from the National Institute on Aging (P01 AG009975) and the National Institute of Diabetes and Digestive and Kidney Diseases (R01 DK127254).

## 40. Enhancing Alertness in Shift Work: Effects of Lighting in Simulated Dispatch Environments

**Author**

Dr. Levent Sahin-United States-Conditioned Light Research & Consultancy

**Abstract**

**Background:** Fatigue among train dispatchers poses a significant risk to operational safety, particularly in the context of growing automation and the increasing centrality of control centers. Traditional countermeasures such as caffeine and naps have shown limited long-term efficacy and practical challenges. A promising alternative involves the application of red light, which can promote acute alertness without suppressing melatonin. However, red light alone may not support visual requirements in occupational environments. This study aimed to evaluate the alerting potential and visual acceptability of a combined red and white light condition compared to red, white, and dim light in a laboratory simulation of a rail dispatch center. **Methods:** Healthy young adults (15 daytime, 16 nighttime participants) were exposed to four lighting conditions: (1) 2700 K white light (50 lux), (2) narrow-band red light (630 nm, 50 lux), (3) a combined red + white light condition (100 lux), and (4) a dim control (<5 lux). Each participant completed EEG recordings, Go/No-Go and Psychomotor Vigilance Tasks (PVT), and rated subjective sleepiness using the Karolinska Sleepiness Scale (KSS). Lighting appraisal questionnaires were also administered to assess perceived comfort and usability. Sessions were conducted separately for daytime and nighttime groups in a counterbalanced design. **Results:** Compared to the dim condition, all active lighting conditions significantly reduced alpha and alpha-theta EEG power, indicating increased alertness. During nighttime, red and combined conditions were particularly effective in reducing alpha power, while white light showed no statistically significant effect. Beta power was generally higher during nighttime, especially at frontal and central EEG sites. Performance measures revealed that participants had significantly faster reaction times and higher scores under the combined condition versus white light, especially after 25 min of exposure. Red light improved hit rates in the Go/No-Go task compared to dim light. Subjective alertness declined over time, but EEG and performance data showed improvements, particularly in later testing blocks. Participants consistently rated the combined lighting condition as more visually comfortable and suitable for office work than red light alone, especially during extended exposure. **Conclusions:** The findings support the use of red and combined (red + white) light to enhance alertness and task performance, particularly during night shifts. The combined condition appears to offer a practical balance by boosting cognitive alertness while maintaining acceptable visual conditions for occupational settings. These results suggest that lighting interventions incorporating red light may be an effective, non-pharmacological strategy to counteract fatigue in rail control environments. Future implementation efforts should involve field evaluations in operational settings aligned with dispatcher schedules to validate and optimize these effects under real-world conditions.

**Keywords:** alertness; red light; circadian health; dispatcher performance; EEG

**Funding:** This work was supported by the National Academies of Sciences, Engineering and Medicine, Transportation Research Board’s Rail Safety IDEA Program.

## 41. Circadian Variation in Metabolic Responses to Meal Timing: Characterizing the 24-Hour Profile of Triglycerides, Remnant Cholesterol, and Glucose in Healthy Adults

**Authors**

Dr. Leilah Grant-United States-1. Division of Sleep and Circadian Disorders, Departments of Medicine and Neurology, Brigham and Women’s Hospital 2. Division of Sleep Medicine, Harvard Medical School

Dr. Kritika Vashishtha-United States-1. Division of Sleep and Circadian Disorders, Departments of Medicine and Neurology, Brigham and Women’s Hospital 2. Division of Sleep Medicine, Harvard Medical School

Dr. Shauni Omond-United States-1. Division of Sleep and Circadian Disorders, Departments of Medicine and Neurology, Brigham and Women’s Hospital

Ms. Lauren McKenzie-United States-Division of Sleep and Circadian Disorders, Departments of Medicine and Neurology, Brigham and Women’s Hospital

Dr. Melissa St. Hilaire-United States-1. Division of Sleep and Circadian Disorders, Departments of Medicine and Neurology, Brigham and Women’s Hospital 2. Division of Sleep Medicine, Harvard Medical School

Dr. Steven Lockley-United States-1. Division of Sleep and Circadian Disorders, Departments of Medicine and Neurology, Brigham and Women’s Hospital 2. Division of Sleep Medicine, Harvard Medical School

Dr. Shadab Rahman-United States-Division of Sleep and Circadian Disorders, Departments of Medicine and Neurology, Brigham and Women’s Hospital; Division of Sleep Medicine, Harvard Medical School

**Abstract**

**Background:** The timing of meals can significantly alter human metabolic responses. For instance, consuming identical meals at night, compared to during the day, acutely raises blood glucose and triglyceride levels. Most prior studies have compared metabolic responses to single meals eaten at two discrete, often opposite, times of day. Therefore, the full 24-h timecourse of metabolic responses to meals remains unknown. This study aimed to systematically examine how meal timing across the 24-h circadian cycle influences daily exposure to triglycerides, triglyceride-rich lipoproteins (remnant-C), and glucose. **Methods:** Ten healthy adults (6 female; age 22–35 years) were studied. Participants completed a 6.5-h eating window that was scheduled during the center of a 16-h intervention day, with the start time randomized to one of 16 stimulus times spaced every 90 min (~22.5°) across the 24-h circadian cycle. Prior to the intervention day, participants completed a 26–48.5-h constant routine (CR), during which they received hourly isocaloric meals. Blood samples were collected every 20–60 min throughout the CR and intervention day and assayed for triglycerides, remnant-C, and glucose. The 24-h area under the curve (AUC) during the intervention day was calculated for each analyte and normalized to the first 24-h AUC from the CR to account for interindividual differences in baseline levels. Relative AUCs were then analyzed using single-harmonic sinusoidal regression to model mealtime response curves across the 24-h cycle. **Results:** Significant 24-h mealtime response curves were observed for triglycerides (*p* < 0.05), remnant-C (*p* < 0.01), and glucose (*p* < 0.001). Triglyceride and remnant-C levels were lowest when the meal window began in the early evening (~7:00 PM), while glucose levels were lowest when the meal window began in the early morning (~9:00 AM). Overall, daily exposure to all three metabolic markers was higher when meals were consumed at night. **Conclusions:** The timing of meals across the 24-h day significantly alters daily exposure to triglycerides, remnant cholesterol, and glucose. These findings indicate that eating during the biological night produces worse metabolic outcomes than eating during the day. This study provides the first continuous 24-h characterization of meal timing effects on key cardiometabolic risk markers and supports the use of meal timing strategies to improve metabolic health. These insights may be especially relevant for shift workers and others who regularly eat during the evening or nighttime hours.

**Support:** Sleep Research Society Career Development Award; R01HL159207.

## 42. Associations Between Personal Light Exposure and Circadian Melatonin Phase in Young Adults with Obsessive-Compulsive Disorder

**Authors**

Dr. Rebecca Cox-United States-Washington University in St. Louis

Ms. Alyssa Week-United States-Washington University in St. Louis

Dr. Kenneth Wright-United States-University of Colorado

**Abstract**

**Background**: Obsessive-compulsive disorder (OCD) is a debilitating psychiatric condition characterized by intrusive thoughts and repetitive behaviors. Although delayed sleep timing and comorbid delayed sleep-wake phase disorder (DSWPD) characterize OCD, factors that contribute to delayed circadian rhythms in OCD are unknown. Light is the primary entrainment cue for the central circadian clock in humans, and unhealthy patterns of personal light exposure are associated with delayed circadian rhythms. We hypothesized that lower intensity, later timing, and more irregular personal light exposure would be associated with later circadian melatonin phase in young adults with OCD. **Methods**: Participants were 38 young adults with OCD (30 female, age_mean_ = 22.18 ± 4.12) and a late bedtime (01:00 or later). Participants completed 2 weeks of free-living ambulatory light exposure (measured in photopic lux) via wrist actigraphy (Actiwatch Spectrum, Phillips Respironics), followed by a 10-h in-laboratory salivary DLMO assessment. Light exposure metrics were calculated using the LightLogR package (0.3.7 version), including time above threshold (TAT) 100, 500, and 1000 lux, mean timing of light exposure (mLIT) above 100, 500, and 1000 lux, intradaily intensity variability, and interdaily intensity stability. DLMO was calculated as the linear interpolated point in time at which melatonin levels rose above threshold (2 SD above the mean of 3 baseline values). Associations between light exposure metrics and DLMO clock hour were examined using univariate and multivariate linear regression models with photoperiod as a covariate. The multivariate model excluded TAT 1000 lux and mLIT 1000 lux due to multicollinearity. **Results**: In univariate models, lower TAT 500 lux, later mLIT 100 lux, mLIT 500 lux, and mLIT 1000 lux, and lower interdaily stability were significantly associated with later DLMO, covarying for photoperiod (*p*’s < 0.05). In the multivariate model, no light metric uniquely predicted DLMO. **Conclusions**: In young adults with OCD and late bedtimes, lower and less stable intensity and later timed personal light exposure were associated with later circadian melatonin phase. No aspect of personal light exposure demonstrated a unique association with circadian melatonin phase, suggesting that multiple aspects of personal light exposure contribute to circadian phase in OCD. Future research is needed to replicate these findings in larger sample sizes using light sensors placed closer to the angle of gaze that measure full-spectrum light exposure.

**Keywords:** light exposure; circadian phase; melatonin; OCD

**Funding:** American Academy of Sleep Medicine Foundation Focused Projects Grant for Junior Investigators; International OCD Foundation Michael A. Yenike Young Investigator Award; NHLBI T32 HL149646.

## 43. Causal Influence of Daytime Sunlight on Sleep Architecture and Next-Day Alertness

**Authors**

Dr. Minqi Yang-China-School of Education, Zhengzhou University, Zhengzhou 450001, China

Prof. Roelof Hut-Netherlands-Department of Chronobiology, University of Groningen, 9712 Groningen, The Netherlands

Dr. Renske Lok-United States-Department of Psychiatry and Behavioral Sciences, Stanford University, Stanford, CA 94305, USA

**Abstract**

**Introduction:** Light serves as the most potent zeitgeber for synchronizing circadian rhythms and shaping sleep-wake cycles. While prior studies have highlighted *associations* between increased daylight exposure and improved sleep quality, causal effects of sunlight exposure on sleep quality and quantity remain largely unexplored in real-world settings. This study aimed to bridge this gap by investigating the impact of a three-hour afternoon stroll under high-intensity light (HIL) or attenuated light (AL) conditions on subjective and objective sleep quality and alertness, providing novel insights into light’s role in regulating human physiology. **Methods:** Ten healthy participants (4 males, 6 females, aged 19–28 years) participated in a within-subject field study. Each participant completed a three-hour afternoon walk, scheduled during the circadian dead zone, under one of two conditions: high-intensity light (HIL, goggles without neutral density filters) or attenuated light (AL, goggles with neutral density filters reducing light intensity to ~10 photopic lux). The conditions were randomized and spaced across two weeks. Salivary Dim Light Melatonin Onset (DLMO) was assessed at baseline and after each walk to determine the circadian phase and adjust the timing of the walk accordingly. Sleep parameters were subsequently monitored using at-home polysomnography (PSG), while subjective alertness and self-reported sleep quality were evaluated with the Karolinska Sleepiness Scale (KSS) and Groningen Sleep Quality Scale (GSQS), respectively. Data were analyzed using linear mixed models to examine changes across conditions. **Results:** The phase shift from baseline to the AL condition was +3 ± 70 min, and from baseline to the HIL condition was −9 ± 42 min, confirming that the walk occurred near the circadian dead zone and that there were no significant differences between interventions in the resulting phase angle of entrainment. No significant differences were found between the HIL and AL conditions in self-selected sleep onset, offset, duration, or subjective sleep quality (all *p*’s > 0.05). However, PSG data revealed a significantly greater number of epochs scored as deep sleep and reduced wakefulness after sleep onset following HIL exposure (all *p*’s < 0.01). Additionally, nighttime delta power accumulation was significantly higher after HIL (*p* < 0.001). Although subjective evening alertness did not differ between conditions (*p* > 0.05), morning alertness was significantly higher following HIL exposure. **Conclusions:** This study demonstrates that daytime exposure to high-intensity light enhances sleep architecture by promoting deeper sleep and reducing lighter sleep stages, while also diminishing next-day sleep inertia. These findings highlight the critical role of daytime light exposure in improving nighttime sleep quality and boosting morning alertness, offering promising applications for alleviating sleep inertia-related challenges.

## 44. Optimizing Tunable Lighting for Human Health

**Authors**

Dr. John Hanifin-United States-Light Research Program, Department of Neurology, Thomas Jefferson University

Mr. Robert Dauchy-United States-Tulane University

Mr. Benjamin Warfield-United States-Light Research Program, Department of Neurology, Thomas Jefferson University

Mr. John Kemp-United States-Light Research Program, Department of Neurology, Thomas Jefferson University

Ms. Lydia Cole-United States-Light Research Program, Department of Neurology, Thomas Jefferson University

Mr. Taehwan Yoo-United States-Light Research Program, Department of Neurology, Thomas Jefferson University

Dr. Robert Karlicek-United States-Rensselaer Polytechnic Institute

Dr. David Blask-United States-Tulane University

Dr. George Brainard-United States-Light Research Program, Department of Neurology, Thomas Jefferson University

**Abstract**

**Background:** The US Department of Energy anticipates that by 2030 LEDs will reach 80% of all lighting sales saving $26 billion per year in electricity costs. The adoption of color tunable LED systems may reduce some of the overall solid-state lighting (SSL) efficiency under the guise of providing improved health and wellness to occupants. There remains, however, a relatively small amount of data regarding the possible positive impacts of daytime tunable SSL lighting on health and wellness as realized in recent research animal studies. To address this gap, studies are needed to quantify characteristics of human metabolic, endocrine and sleep physiology affected by tunable SSL versus typical fluorescent lighting. **Methods:** The goal of this project is to test the efficacy of tunable SSL inclusive of bright short wavelength enriched SSL versus dimmer, fixed spectrum cool white fluorescent (CWF) lighting on a range of metabolic (glucose, insulin, leptin), endocrine (cortisol, melatonin) and sleep (onset, duration, efficiency) physiology in healthy humans. Two studies are underway testing the hypothesis that, compared to dimmer, commonly used CWF lighting, daytime tunable lighting inclusive of bright short wavelength enriched SSL and dimmer, short wavelength depleted SSL will improve participants’ overall health as measured by metabolic, endocrine and sleep physiology. One study (*n* = 12, crossover) is being performed over 7 days in a controlled laboratory setting. The other study will include more natural conditions. The 9-day naturalistic study (*n* = 28) will have a hybrid of controlled exposure to SSL during an 8-h daytime work period, followed by subjects leaving the laboratory to receive light exposures in their chosen public and domestic settings. We hypothesize that, compared to static, dimmer, daily lighting of CWF lamps tunable bright short wavelength enriched SSL will increase the amplitude and duration of melatonin production, advance onset of melatonin secretion, optimize glucose, insulin, leptin and cortisol levels, shorten sleep latency and improve sleep efficiency. Both studies are intended to add evidence relevant to the purported benefits of tunable SSL in indoor lighting environments. **Results:** Seven participants have completed both arms of the controlled laboratory study. Data collection and analysis are ongoing. **Conclusions:** The goal of this project is to characterize the link between daytime lighting and human health for users of SSL tunable lighting system using quantified endpoints of metabolism, endocrine function, and sleep physiology.

**Keywords:** light; sleep; melatonin; cortisol; glucose; insulin; leptin; solid state lighting

**Support:** Primary funding: DOE Agreement #DE-EE0009689.

## 45. Longitudinal Associations Between Circadian Rhythms and Cancer-Related Symptoms in Breast Cancer Patients

**Authors**

Dr. Lisa Wu-Iceland-Sleep & Circadian Psychology Research Unit, Department of Psychology and Behavioural Sciences, Aarhus University, and Department of Psychology, Reykjavik University, Reykjavik, Iceland

Dr. Ru Li-Denmark-Sleep & Circadian Psychology Research Unit, Department of Psychology and Behavioural Sciences, Aarhus University, Aarhus, Denmark

Dr. Dinne Christensen-Denmark-Department of Psychology & Behavioral Sciences, Aarhus University

Prof. Robert Zachariae-Denmark-Unit for Psychooncology & Health Psychology, Aarhus University Hospital

Prof. Peer Christiansen-Denmark-Department of Plastic and Breast Surgery, Aarhus University Hospital

Prof. Anders Bonde-Denmark-Department of Clinical Medicine, Aarhus University

Prof. Frank Penedo-United States-Departments of Psychology and Medicine, University of Miami Miller School of Medicine

Prof. Sonia Ancoli-Israel-United States-Department of Psychiatry, University of California San Diego School of Medicine

Prof. Kathryn Reid-United States-Center for Circadian and Sleep Medicine, Northwestern University, Chicago, USA

Dr. Ali Amidi-Denmark-2. Sleep & Circadian Psychology Research Unit, Department of Psychology and Behavioural Sciences, Aarhus University, Aarhus, Denmark.

**Abstract**

**Background:** Breast cancer (BC) patients frequently experience a range of behavioral and psychological symptoms throughout the cancer trajectory, including fatigue, sleep disturbances, depression, and cognitive complaints. These cancer-related symptoms (CRS) may emerge prior to treatment, worsen during treatment, and persist well beyond its completion, significantly impairing quality of life. Increasing evidence suggests that circadian rhythm disruption may contribute to the pathophysiology of CRS. This study aimed to prospectively examine changes in circadian rhythm markers and CRS from diagnosis to 12 months later in recently diagnosed BC patients, compared with a matched healthy control group. *Trial registration: NCT04401189.* **Methods:** Sixty-five newly diagnosed BC patients were enrolled prior to any cancer treatment. Assessments were conducted at four time points: Baseline (T1, pre-treatment), post-surgery or neoadjuvant chemotherapy (T2), post-adjuvant chemotherapy and/or surgery (T3), and one-year follow-up (T4). Sixty-nine age-matched healthy controls (HCs) were assessed at parallel time points at T1, T3 and T4. Only data from T1, T3, and T4 were included in the present analyses. Circadian activity rhythms were measured using seven-day actigraphy, from which the Circadian Function Index (CFI) was calculated. Dim-light melatonin onset (DLMO) was assessed in patients at T1 and T4 via seven hourly saliva samples. Fatigue, insomnia, sleep quality, depressed mood and cognitive complaints were measured using the FACIT-Fatigue, Insomnia Severity Index, Pittsburgh Sleep Quality Index, Center for Epidemiologic Studies—Depression Scale, and the FACT-Cog respectively. Multi-group latent growth curve modeling was used to examine symptom trajectories and to evaluate the predictive effects of CFI and DLMO on these trajectories among patients. **Results:** Compared to HCs, BC patients showed significantly greater increases in fatigue (*p* = 0.011) and cognitive complaints (*p* < 0.001) over time, while depressed mood showed a significant decline (*p* = 0.027). Sleep quality and insomnia were significantly worse in patients at baseline (*p* < 0.001 for both), but their trajectories were similar across groups. DLMO advanced by an average of 0.78 h from T1 to T4 (*p* = 0.003). The greater this advance, the greater were the increases in fatigue (lower score worse; β = −0.314, *p* = 0.048) and the smaller were the reductions in depressed mood (higher score worse; β = 0.401, *p* = 0.024). Although CFI remained stable across time points and groups, higher average CFI across time was associated with more favorable trajectories—predicting smaller increases in fatigue (β = 0.317, *p* = 0.049) and greater reductions in depressed mood (β = −0.538, *p* = 0.025). **Conclusions:** This study highlights the role of circadian dysregulation in the development and persistence of cancer-related symptoms in BC patients. Advances in melatonin timing and less robust circadian activity rhythms were associated with worse trajectories of fatigue and depressed mood, suggesting that circadian disruptions may contribute to the maintenance of CRS over time. These findings underscore the importance of considering circadian rhythms in symptom monitoring and point to potential avenues for chronotherapeutic interventions aimed at alleviating long-term symptom burden among breast cancer survivors.

**Keywords:** circadian rhythms; breast cancer; cancer survivorship; fatigue; depression; actigraphy; melatonin

**Funding:** Danish Cancer Society #R174-A11447-17-S52.

## 46. Rise, Shine, and Simulate: An Intro to Circadian Modelling

**Author**

Dr. Melissa St Hilaire-United States-Department of Computer and Data Sciences, School of Engineering and Computational Sciences, Merrimack College, North Andover, MA

**Abstract**

Circadian rhythms are endogenously generated, entrainable oscillations that regulate a wide range of physiological and behavioral processes with a near-24-h period. Accurate modeling of these rhythms is essential for understanding circadian phase, amplitude modulation, and entrainment dynamics, especially in applied contexts such as shift work, jet lag, and chronotherapeutics. This session provides a methodological overview of two major approaches to circadian modelling. We begin with cosinor analysis, a parametric method for fitting sinusoidal models to rhythmic time-series data. Cosinor models estimate mesor (i.e., rhythm-adjusted mean), amplitude (i.e., oscillatory strength), and acrophase (i.e., timing of peak), and can be extended to multi-component or population-mean cosinor models. We will review statistical considerations (e.g., confidence intervals, goodness-of-fit), demonstrate open-source implementations (e.g., cosinor2 in R, CosinorPy in Python), and highlight use cases in physiological signal analysis. We then transition to mechanistic circadian models—dynamical systems designed to simulate the phase and amplitude of the internal biological clock as a function of light exposure and behavioral schedules—focusing specifically on the Kronauer family of van der Pol-type oscillator models. We will examine model parameters, assumptions and outputs, and demonstrate how these models estimate circadian phase under real-world light-dark and sleep-wake inputs. This session therefore will provide a rigorous overview of methods for estimating circadian phase and rhythm parameters from observational data and simulated sleep-wake and light inputs. Emphasis will be placed on transparent modeling practices and the interpretation of outputs from both statistical and dynamical frameworks. By comparing and contextualizing cosinor- and oscillator-based approaches, the session will highlight how model-derived metrics can support phase estimation, assess circadian misalignment, and guide experimental design in chronobiology.

## 47. Sleep Duration Is Associated with the Timing of Sleep Onset, Not the Duration of Prior Wakefulness: An Analysis of ~450,000 Sleep-Wake Episodes

**Authors**

Dr. Angus Burns-United States-Division of Sleep and Circadian Disorders, Departments of Medicine and Neurology, Brigham and Women’s Hospital; Division of Sleep Medicine, Harvard Medical School

Dr. Daniel Windred-Australia-Flinders Health and Medical Research Institute: Sleep Health, Flinders University, Adelaide, Australia

Dr. Richa Saxena-United States-Division of Sleep and Circadian Disorders, Departments of Medicine and Neurology, Brigham and Women’s Hospital; Division of Sleep Medicine, Harvard Medical School; Department of Neurology, Massachusetts General Hospital

Prof. Frank Scheer-United States-Division of Sleep and Circadian Disorders, Departments of Medicine and Neurology, Brigham and Women’s Hospital

Dr. Charles Czeisler-United States-Division of Sleep and Circadian Disorders, Departments of Medicine and Neurology, Brigham and Women’s Hospital; Division of Sleep Medicine, Harvard Medical School

Dr. Sean Cain-Australia-Flinders Health and Medical Research Institute: Sleep Health, Flinders University, Adelaide, Australia

Dr. Andrew Phillips-Australia-Flinders Health and Medical Research Institute: Sleep Health, Flinders University, Adelaide, Australia

Dr. Jackie Lane-United States-Division of Sleep and Circadian Disorders, Departments of Medicine and Neurology, Brigham and Women’s Hospital

**Abstract**

**Background:** Sleep duration is jointly regulated by the circadian pacemaker and a homeostatic process. Yet, classical temporal isolation experiments have repeatedly made a counterintuitive observation: longer episodes of wakefulness (α) tend to be followed by shorter episodes of sleep (ρ). This leads to a negative α:ρ correlation. These laboratory studies have shown that the circadian gating of the sleep-wake cycle explains this negative correlation and, most strikingly, the residual α:ρ correlation has been observed to be ~0. This observation seemingly contradicts traditional restorative hypotheses of sleep; however, no study has tested if this phenomenon generalizes to free-living humans living in real-world settings. **Methods:** To examine this, we analyzed ~450,000 sleep-wake episodes from 85,955 free-living older adults (62.3 ± 7.9 years, 58% female), using wearable-recorded light exposure and sleep data. Individual circadian phase (timing of core-body temperature minimum, CBTmin) was estimated using a validated model of the response of the central circadian pacemaker to light. **Results:** We observed that longer prior wake duration was negatively associated with subsequent sleep duration (α:ρ β = –0.42, *p* < 5 × 10^−16^), such that a one-hour longer wake episode predicted a 0.42-h shorter subsequent sleep episode. Sleep duration exhibited marked variation with the predicted circadian phase of sleep onset: sleep episodes initiated at −8, −4, 0, and 4 h relative to CBTmin were 10.23, 7.53, 5.06, and 3.13 h in duration, respectively (*p* < 5 × 10^−16^). These relationships were independent of age, sex and employment. Notably, after accounting for the predicted circadian phase of sleep onset, the association between prior wake duration and sleep duration was substantially attenuated (α:ρ β = −0.001, *p* = 0.63). **Conclusions:** As in temporal isolation studies, we observed that longer wakefulness predicted shorter subsequent sleep duration and that sleep duration depended on the estimated circadian phase of sleep onset. We also observed that after accounting for the circadian phase of sleep onset there is only a very weak association between prior wakefulness and sleep duration. Taken together, our findings provide evidence that the circadian gating of sleep duration also operates in naturalistic environments and challenge the traditional restorative hypotheses of sleep.

## 48. Building on Our Discoveries

**Authors**

Dr. Janis Anderson-United States-Corresponding Member of the Faculty of Psychology, Department of Psychiatry, Harvard Medical School; Scholar, Brigham & Women’s Hospital, Boston, MA and Division of Sleep Medicine, Harvard Medical School, Boston, MA

Dr. Rana Sagha Zadeh-United States-Associate Professor; Director, Health Design Innovations Lab, Cornell University

**Abstract**

Calls for sunlight in hospital wards date back centuries—Florence Nightingale advocated for including windows in the 1800s [1]. In the USA, formal standards for hospital design emerged in 1947 and have steadily evolved. By the late 20th century, healthcare design, engineering, and management began adopting a more data-driven approach inspired by Evidence-based Medicine. Known as Evidence-based Design (EBD), it integrates data on clinical outcomes into decision making as design options are evaluated in the planning of new construction. Relying on outcomes data showing enhanced health/patient outcomes and operational performance (Hamilton, 2003; Stichler & Hamilton, 2008) [2,3]. EBD has revealed measurable effects of the built environment on health and healing, staff efficiency, and safety (Ulrich et al., 2004) [4]. Although medical science and technological innovation are rapidly advancing, the translation into practice remains slow. To narrow the gap, a novel framework known as Evidence-Based and Value-Based Design has been introduced by Zadeh et al. [5]. This framework emphasizes not only measured clinical outcomes, but also demonstrable economic returns calculated from performance data such as reduced injuries and improved retention of staff, along with enhanced recovery and satisfaction of patients. Healthcare facilities are inherently complex, requiring multiple clinical and non-clinical professionals. The Evidence-based and Value-based Design framework comes out of a Systems Approach [6] that engages the multidisciplinary partnerships required to provide excellent healthcare. This framework aligns with translational research models (e.g., NIH’s 5-stage CTRIS framework), emphasizing continued interdisciplinary collaboration in design, build, implementation and evaluation. We suggest that by computing the financial Health Outcome Benefits of innovations such as Dynamic Lighting, Ref. [7] Value-Based Design invites the participation of health administrators and brings financial transparency to decisions that affect health outcomes. The economics of improved health outcomes include reduced operational, business, and organizational costs. Value-based analyses demonstrate how strategic investments in healthcare environments can lead to tangible outcomes such as improved staff retention, better performance, reduced errors and staff injuries, as well as decreased patient pain, faster recovery, and increased patient satisfaction. Value-based analyses communicate in financial terms the return on investment that modifications such as Dynamic Lighting can provide, so that science-based innovations will not be eliminated from facilities planning based on the false assumption that they are nice in theory, but unaffordable in practice. SLRCH, as a Society of clinicians and scientists who aim to further improve human health, may strengthen our effectiveness by systematically collecting evidence documenting in the broadest terms the value of health benefits our interventions produce. The Evidence-based and Value-based framework provides one tool we can employ in order to make our case in terms our partners in other key roles readily appreciate. Through stronger collaboration across disciplines and with stakeholders, SLRCH members have the opportunity to not only generate scientific knowledge, but also help translate it into better, safer, and more effective places of care.

**References**

1. Zborowsky, T. The Legacy of Florence Nightingale’s Environmental Theory: Nursing Research Focusing on the Impact of Healthcare Environments. *HERD* **2014**, *7*, 19–34. https://doi.org/10.1177/193758671400700404. PMID: 25303425.
2. Hamilton, D.K. The Four Levels of Evidence-based Practice. *Healthc. Des.* **2003**, *3*, 18–26.
3. Stichler, J.F.; Hamilton, D.K. Evidence-based design: what is it? *HERD* **2008**, *1*, 3–4. https://doi.org/10.1177/193758670800100201. PMID: 21161891.
4. Ulrich, R.S.; Zimring, C.; Zhu, X.; DuBose, J.; Seo, H.B.; Choi, Y.S.; Quan, X.; Joseph, A. A review of the research literature on evidence-based healthcare design. *HERD* **2008**, *1*, 61–125. https://doi.org/10.1177/193758670800100306. PMID: 21161908.
5. Zadeh, R.; Sadatsafavi, H.; Xue, R. Evidence-Based and Value-Based Decision Making About Healthcare Design: An Economic Evaluation of the Safety and Quality Outcomes. *HERD* **2015**, *8*, 58–76. https://doi.org/10.1177/1937586715586393. PMID: 26123968.
6. Ezra’s Round Table Systems Seminar. YouTube Cornell Systems Engineering Archive. Available online: https://youtu.be/ovdOSlOpLPI?si=iVenWZbAueoPeMji (accessed on 30 October 2020).
7. Grant, L.K.; St Hilaire, M.A.; Heller, J.P.; Heller, R.A.; Lockley, S.W.; Rahman, S.A. Impact of Upgraded Lighting on Falls in Care Home Residents. *J. Am. Med. Dir. Assoc*. **2022**, *23*, 1698–1704.e2. https://doi.org/10.1016/j.jamda.2022.06.013. PMID: 35850166.

## 49. Sleep and Circadian Genetic Clusters Reveal Divergent Clinical Profiles in Bipolar Disorder: Evidence from Phenome-Wide Association and Trait Characterization

**Authors**

Dr. Lovemore Kunorozva-United States-Division of Sleep and Circadian Disorders, Departments of Medicine and Neurology, Brigham and Women’s Hospital

Dr. Susan Redline-United States-Division of Sleep and Circadian Disorders, Departments of Medicine and Neurology, Brigham and Women’s Hospital

Mr. Jesse T Valliere-United States-Department of Anesthesia, Critical Care, and Pain Medicine, Massachusetts General Hospital; Division of Sleep Medicine, Harvard Medical School

Dr. Anastasia Yocum-United States-Department of Psychiatry, University of Michigan Medical School

Dr. Hellen Burgess-United States-Department of Psychiatry, University of Michigan Medical School, Rachel Upjohn Building, 4250 Plymouth Road, Ann Arbor, MI 48109

Dr. Sarah Sperry-United States-Department of Psychiatry, University of Michigan Medical School

Prof. Richa Saxena-United States-Department of Anesthesia, Critical Care, and Pain Medicine, Massachusetts General Hospital; Division of Sleep Medicine, Harvard Medical School

Dr. Jackie Lane-United States-Division of Sleep and Circadian Disorders, Departments of Medicine and Neurology, Brigham and Women’s Hospital

**Abstract**

**Background:** Sleep and circadian rhythm disruption is a core feature of bipolar disorder (BD), influencing disease onset, symptom severity, and treatment outcomes. Despite strong evidence for heritability of these traits, the underlying genetic pathways that connect sleep and circadian rhythms to the clinical heterogeneity in BD remain poorly defined. Polygenic risk scores (PRS) for sleep and circadian phenotypes can be used to explore genetic subtypes within BD, but few studies have evaluated how such PRS clusters relate to clinical outcomes. Here, we used a data-driven clustering approach to identify distinct genetic sleep and circadian profiles and investigated their phenotypic associations using a phenome-wide association study (PheWAS) framework. **Methods:** We applied Bayesian Non-negative Matrix Factorization (bNMF) to group previously identified 63-BD risk variants (Mullin et al. 2021) [1] by their association with 17 sleep and circadian traits (10 self-reported, 7 actigraphy-derived) from the UK Biobank, aligning effects to the BD risk increasing variant. Only traits that passed the trait filtering criteria (n = 10) defined as having a Bonferroni—corrected p-value below 0.05 divided by the number of tested SNPs were retained for clustering. The bNMF model decomposed the matrix of BD-variant-by-trait associations (X) into two low-rank matrices W and H, such that X ≈ WH, where the optimal number of clusters (K) was inferred through automatic relevance determination. The resulting clusters were characterized based on their top-weighted genetic variants and phenotypic traits. We computed PRS for each cluster and conducted PheWAS in an independent dataset to examine associations with clinical diagnoses using the MassGeneralBrigham cohort. Models were adjusted for ancestry, sex, and age, and false discovery rate (FDR) correction was applied for multiple testing. **Results:** Two robust clusters emerged: *Cluster 1* reflected a late chronotype, shorter time in bed, increased daytime sleepiness, frequent naps, and more insomnia symptoms; *Cluster 2* reflected an early chronotype, longer sleep duration, and fewer insomnia symptoms. *Cluster 1* PRS was significantly associated with neurodevelopmental and psychiatric diagnoses including attention-deficit/hyperactivity disorder (OR = 1.08, *p* = 8.6 × 10^−5^), pervasive developmental disorders, psychosis, and impulse control disorders. These associations align with the cluster’s phenotype of increased sleep disturbance and circadian misalignment. In contrast, *Cluster 2* PRS was associated with cardiometabolic outcomes such as disorders of carbohydrate metabolism (OR = 1.06, *p* = 1.1 × 10^−4^) and aortic valve disease (OR = 0.83, *p* = 0.002), indicating a potential protective or distinct physiological profile. The divergent sleep trait profiles and clinical associations across clusters suggest unique etiologic pathways within BD. **Conclusions:** This study identifies biologically and clinically distinct genetic clusters of sleep and circadian rhythm traits in BD using a bNMF approach. The distinct clinical profiles, psychiatric in Cluster 1 *vs*. metabolic in Cluster 2, underscore the utility of PRS clustering in stratifying BD-patients for tailored interventions. These findings provide a framework for integrating genetic sleep architecture into precision psychiatry and advance our understanding of pleiotropic mechanisms linking sleep, circadian disruption, and BD.
  **Background:** Sleep and circadian rhythm disruption is a core feature of bipolar disorder (BD), influencing disease onset, symptom severity, and treatment outcomes. Despite strong evidence for heritability of these traits, the underlying genetic pathways that connect sleep and circadian rhythms to the clinical heterogeneity in BD remain poorly defined. Polygenic risk scores (PRS) for sleep and circadian phenotypes can be used to explore genetic subtypes within BD, but few studies have evaluated how such PRS clusters relate to clinical outcomes. Here, we used a data-driven clustering approach to identify distinct genetic sleep and circadian profiles and investigated their phenotypic associations using a phenome-wide association study (PheWAS) framework. **Methods:** We applied Bayesian Non-negative Matrix Factorization (bNMF) to group previously identified 63-BD risk variants (Mullin et al. 2021) [1] by their association with 17 sleep and circadian traits (10 self-reported, 7 actigraphy-derived) from the UK Biobank, aligning effects to the BD risk increasing variant. Only traits that passed the trait filtering criteria (n = 10) defined as having a Bonferroni—corrected p-value below 0.05 divided by the number of tested SNPs were retained for clustering. The bNMF model decomposed the matrix of BD-variant-by-trait associations (X) into two low-rank matrices W and H, such that X ≈ WH, where the optimal number of clusters (K) was inferred through automatic relevance determination. The resulting clusters were characterized based on their top-weighted genetic variants and phenotypic traits. We computed PRS for each cluster and conducted PheWAS in an independent dataset to examine associations with clinical diagnoses using the MassGeneralBrigham cohort. Models were adjusted for ancestry, sex, and age, and false discovery rate (FDR) correction was applied for multiple testing. **Results:** Two robust clusters emerged: *Cluster 1* reflected a late chronotype, shorter time in bed, increased daytime sleepiness, frequent naps, and more insomnia symptoms; *Cluster 2* reflected an early chronotype, longer sleep duration, and fewer insomnia symptoms. *Cluster 1* PRS was significantly associated with neurodevelopmental and psychiatric diagnoses including attention-deficit/hyperactivity disorder (OR = 1.08, *p* = 8.6 × 10^−5^), pervasive developmental disorders, psychosis, and impulse control disorders. These associations align with the cluster’s phenotype of increased sleep disturbance and circadian misalignment. In contrast, *Cluster 2* PRS was associated with cardiometabolic outcomes such as disorders of carbohydrate metabolism (OR = 1.06, *p* = 1.1 × 10^−4^) and aortic valve disease (OR = 0.83, *p* = 0.002), indicating a potential protective or distinct physiological profile. The divergent sleep trait profiles and clinical associations across clusters suggest unique etiologic pathways within BD. **Conclusions:** This study identifies biologically and clinically distinct genetic clusters of sleep and circadian rhythm traits in BD using a bNMF approach. The distinct clinical profiles, psychiatric in Cluster 1 *vs*. metabolic in Cluster 2, underscore the utility of PRS clustering in stratifying BD-patients for tailored interventions. These findings provide a framework for integrating genetic sleep architecture into precision psychiatry and advance our understanding of pleiotropic mechanisms linking sleep, circadian disruption, and BD.
**Keywords:** bipolar disorder; polygenic risk score; sleep; circadian rhythms; PheWAS; precision psychiatry
**References**

1. Mullins, N.; Forstner, A.J.; O’Connell, K.S.; Coombes, B.; Coleman, J.R.I.; Qiao, Z.; Als, T.D.; Bigdeli, T.B.; Børte, S.; Bryois, J. Genome-wide association study of over 40,000 bipolar disorder cases provides new insights into the underlying biology. *Nat Genet.* **2021**, *53*, 817–829. https://doi.org/10.1038/s41588-021-00857-4.

## 50. Associations Between Light Exposure and Cognitive Performance in the Multi-Ethnic Study of Atherosclerosis (MESA)

**Authors**

Ms. Runpeng Hu-United States-Harvard T.H. Chan School of Public Health

Dr. Susan Redline-United States-Division of Sleep and Circadian Disorders, Departments of Medicine and Neurology, Brigham and Women’s Hospital

**Abstract**

**Introduction**: Light is essential for vision but also exerts non-image-forming (NIF) effects on human physiology and behavior. These effects are mediated by intrinsically photosensitive retinal ganglion cells (ipRGCs), which express melanopsin and project to the suprachiasmatic nuclei (SCN), the brain’s central circadian clock. In modern environments, artificial lighting and irregular exposure patterns may disrupt circadian rhythms and contribute to cognitive decline. While the effects of light on sleep are well established, its direct relationship with cognitive outcomes remains underexplored in large, diverse populations. This study investigates whether white light exposure is associated with cognitive performance in older adults from the Multi-Ethnic Study of Atherosclerosis (MESA). **Methods**: The analytic sample included 1711 participants (mean age: 68.4 years; 54.4% female) from the MESA Sleep Ancillary Study (2010–2013) with valid wrist actigraphy recordings, with demographic and cognitive data from MESA Exam 5. White light exposure was measured in 30-s epochs using Actiwatch devices (Philips Respironics, Bend, OR, USA), and various light metrics were calculated from the raw data for analysis. The primary exposure was the log10-transformed average daily illuminance. Secondary exposures included duration above 1000 lux (TALT1000) and average daytime and nighttime illuminance, aligned with each individual’s sleep schedule. The primary cognitive outcome was the Digit Symbol Coding (DSC) test score. Covariates included sociodemographic characteristics, cardiovascular and health risk factors, physical activity, depression, sleep duration and irregularity, as well as study site and seasonality. We used a tiered multivariable linear regression approach to evaluate associations and conducted sensitivity analyses to explore potential effect modification and robustness of results. **Results**: Greater average log10 illuminance was significantly associated with higher DSC scores in unadjusted models (β = 8.09; 95% CI: 5.20–10.98; *p* < 0.001). This association remained robust and statistically significant after adjusting for sociodemographic, site, seasonal, behavioral, and cardiovascular factors (β ≈ 3.93–4.13; all *p* < 0.05). Even after adjusting for sleep duration and irregularity this association remained statistically significant. TALT1000 showed a positive association in unadjusted models but lost significance after adjustment. Daytime light exposure was positively associated with DSC scores, while nighttime light showed a negative association; however, neither remained significant after adjustment for sleep metrics. Sensitivity analyses confirmed the overall pattern, though associations were somewhat sensitive to sleep variables. **Conclusions**: We found that habitual daily light exposure is positively associated with cognitive performance in older adults. Average light intensity had more consistent associations than duration of bright light exposure. Timing also appeared important: greater daytime and morning light exposure showed positive trends, while nighttime light may have negative effects. However, these relationships may be partly mediated or confounded by sleep characteristics. Future studies should prioritize temporally aligned measures of light and cognition, and further investigate the mechanistic role of circadian regulation. These findings support the potential of optimizing ambient light environments to promote cognitive health in aging populations.

## 51. Objective Prediction of Siesta Based on Machine Learning and Association with Obesity

**Authors**

Ms. Maria Rodriguez-Spain-Department of Physiology, University of Murcia

Mr. Fernando Moreno-Caballero-Spain-Department of Informatics and Systems, University of Murcia

Dr. Hassan S Dashti-United States-Department of Anesthesia, Critical Care, and Pain Medicine, Massachusetts General Hospital; Division of Sleep Medicine, Harvard Medical School

Prof. Richa Saxena-United States-Department of Anesthesia, Critical Care, and Pain Medicine, Massachusetts General Hospital; Division of Sleep Medicine, Harvard Medical School

Prof. Frank Scheer-United States-Division of Sleep and Circadian Disorders, Departments of Medicine and Neurology, Brigham and Women’s Hospital

Prof. Jesualdo T Fernández-Breis-Spain-Department of Informatics and Systems, University of Murcia

Prof. Marta Garaulet-Spain-Department of Physiology, University of Murcia

**Abstract**

**Background.** Obesity is a growing global health concern, projected to affect 51% of the world population by 2035. Lifestyle factors, including sleep hygiene, play a key role in its prevention. Previous studies have linked long siesta episodes with increased metabolic risk, particularly in Mediterranean and UK populations. The integrative TAP variable, combining temperature (T), activity (A), and position (P), has shown promise in objective sleep assessment, though its use in siesta prediction remains limited. While machine learning (ML) has been applied to sleep disorders, it has yet to be used to capture everyday siesta patterns. We aim to objectively predict siesta behavior using ML based on T, A, P, and the integrative TAP variable, and to investigate associations between objectively measured siesta characteristics and obesity-related traits in a Mediterranean population. **Methods.** A total of 889 healthy adults from the ONTIME-MT study wore wrist-worn sensors continuously for 7 days to capture T, A, and P data. Participants also self-reported their daily siesta behavior. All variables were compiled every 30 s, and TAP was computed by integrating the three signals. Data smoothing was performed using a moving average technique, and missing values were imputed via K-nearest neighbors. Multiple ML models were trained to classify siesta vs. non-siesta intervals, with a decision tree model showing the best balanced accuracy. Anthropometric and metabolic markers including BMI, fat percentage, waist circumference (WC), blood pressure, and glucose metabolism (assessed via 2-h Oral Glucose Tolerance Test) were measured. Linear and logistic regressions were conducted to assess associations between siesta timing, duration, and frequency and obesity/metabolic traits. **Results.** ML models achieved an 83% success rate in predicting siesta episodes. The decision tree revealed that activity was the most informative predictor, with a threshold of 27 Δ°/min differentiating siesta (41%) from non-siesta (59%) intervals. TAP and position contributed further discrimination, with thresholds of 0.51 arbitrary units and 4.7°, respectively. Objectively predicted siesta, but not self-reported, was significantly associated with various obesity-related outcomes. Specifically, later siesta timing was linked to higher WC in women (Beta = 0.769 cm per 1-h delay; *p* = 0.026). Longer siesta duration was associated with increased odds of obesity (OR = 2.081; *p* = 0.002), higher BMI (Beta = 0.013 kg/m^2^ per 1-h increase; *p* = 0.034), and elevated systolic blood pressure (Beta = 3.540 mmHg per 1-h increase; *p* = 0.049). Additionally, more frequent siesta was correlated with impaired corrected insulin response (Beta = −0.037 per additional siesta day; *p* = 0.012). On average, participants overestimated siesta duration by approximately 10 min when self-reporting. **Conclusions.** This study demonstrates that ML techniques utilizing T, A, P, and the integrative TAP variable can objectively and accurately predict siesta behavior in free-living conditions. Notably, only objectively assessed siesta was significantly associated with obesity-related traits. Our findings support the relevance of siesta characteristics in metabolic health and emphasize the need for standardized, objective approaches to siesta evaluation. Future studies should validate these associations in other populations and assess their potential for clinical or preventive applications.

## 52. The Future of Circadian Research: Evaluating Chat GPT’s Ability to Distill Data on the Diagnosis & Treatment of Advanced and Delayed Sleep-Wake Phase Disorder

**Authors**

Ms. Ebele Okafor-United States-Division of Sleep and Circadian Disorders, Departments of Medicine and Neurology, Brigham and Women’s Hospital

Dr. Lovemore Kunorozva-United States-Division of Sleep and Circadian Disorders, Departments of Medicine and Neurology, Brigham and Women’s Hospital

Dr. Jacqueline Lane-United States-Division of Sleep and Circadian Disorders, Departments of Medicine and Neurology, Brigham and Women’s Hospital

**Abstract**

**Background:** Circadian rhythm sleep-wake disorders are a misalignment between one’s sleep and the biosocial 24-h cycle. Advanced/Delayed sleep-wake phase disorder (ASWPD/DSWPD) are challenging to treat as patients experience a lack of resources and are often misdiagnosed, with ~10% of insomnia patients having DSWPD. This systematic review (1) summarized the diagnostic and treatment tools for ASWPD/DSWPD; (2) examined study definitions for ASWPD/DSWPD; (3) evaluated study quality; and (4) identified gaps in knowledge. **Methods:** We screened 1805 records using the 2020 Preferred Reporting Items for Systematic Reviews and Meta-Analyses (PRISMA). We also tested *ChatGPT-4.0‘s* ability to extract and summarize data, using *BLEU* and *ROUGE* metrics to grade the AI’s precision and recall. **Results:** Our review identified 49 published articles, spanning over 20 years. The majority used actigraphy, sleep logs, and surveys for diagnosis. Treatment mainly consisted of melatonin, light therapy, and cognitive-behavioral therapy. *ChatGPT-4.0* was accurate about 75% of the time in extracting study data and simplified the review process. **Conclusions:** This review identified a general set of diagnostic criteria for ASWPD/DSWPD, and a need for additional studies of ASWPD and data across the lifespan. *ChatGPT’s* data extraction will be a beneficial asset in medical chart review, and with this library of literature, aid in combating misdiagnosis.

## 53. Shift and Longtime Light Induce Endometrioid Adenocarcinoma in the Female Golden Hamster

**Author**

Dr. Sanjeev Yadav-India-Department of Zoology, Faculty of Science, Banaras Hindu University, Varanasi 221 005, India

**Abstract**

Female night workers get exposed to frequent light shifts, hence have altered circadian rhythm, and are at high risk of endometrial cancer; the underlying mechanism, however, is still not clear. We, therefore, examined the effect of long light exposure (16L:8D, LD1) and regular shift (8 h) in long nighttime (LD2) on endometrial changes in female golden hamsters. Morphometric analysis, scanning electron microscopy imaging, alcian blue staining, and cytological nuclear atypia of endometrial stromal cells confirmed the incidence of endometrial adenocarcinoma in LD2 exposed hamsters. However, less severe pathomorphological alterations were noted in the uterus of LD1-exposed hamsters. Altered Aanat and Bmal1 mRNA, melatonin rhythm, downregulation of important marker genes of adenocarcinoma like Akt, 14-3-3, and PR protein expression and upregulation PKCα, pAkt-S473 and vascular epithelial growth factor (VEGF) were observed in LD2 exposed hamsters suggesting the endometrial adenocarcinoma. Overall, our data indicates that light shift and long light exposure potentially induced endometrioid adenocarcinoma via activation of the PKC-α/Akt pathway in female hamsters. Therefore, the duration of light is essential for normal female uterine function.

